# Metal Oxide Nanocatalysts for the Electrochemical Detection of Propofol

**DOI:** 10.3390/mi16020120

**Published:** 2025-01-22

**Authors:** David C. Ferrier, Janice Kiely, Richard Luxton

**Affiliations:** Institute of Bio-Sensing Technology, University of the West of England, Frenchay Campus, Bristol BS16 1QY, UK; david.ferrier@uwe.ac.uk (D.C.F.); janice.kiely@uwe.ac.uk (J.K.)

**Keywords:** metal oxide nanoparticle, propofol, electrochemical sensor, nanocomposite, graphene oxide, green synthesis

## Abstract

Propofol is one of the most widely used intravenous drugs for anaesthesia and sedation and is one of the most commonly used drugs in intensive care units for the sedation of mechanically ventilated patients. The correct dosage of propofol is of high importance, but there is currently a lack of suitable point-of-care techniques for determining blood propofol concentrations. Here, we present a cytochrome P450 2B6/carbon nanotube/graphene oxide/metal oxide nanocomposite sensor for discrete measurement of propofol concentration. Propofol is converted into a quinol/quinone redox couple by the enzyme and the nanocomposite enables sensitive and rapid detection. The metal oxide nanoparticles are synthesised via green synthesis and a variety of metal oxides and mixed metal oxides are investigated to determine the optimal nanocatalyst. Converting propofol into the redox couple allows for the measurement to take place over different potential ranges, enabling interference from common sources such as paracetamol and uric acid to be avoided. It was found that nanocomposites containing copper titanium oxide nanoparticles offered the best overall performance and electrodes functionalised with such nanocomposites demonstrated a limit of detection in bovine serum of 0.5 µg/mL and demonstrated a linear response over the therapeutic range of propofol with a sensitivity of 4.58 nA/μg/mL/mm^2^.

## 1. Introduction

Nanomaterials are frequently used in electrochemical sensing and biosensing applications because of their high surface area/volume ratios, excellent electron transfer characteristics and potential catalytic behaviour [1,2]. Metal oxide nanoparticles are commonly used, particularly those incorporating transition metals, due to their low cost, chemical stability and biocompatibility [3,4,5,6]. In addition, they show good catalytic behaviour as a result of the partially filled d-shells of the metals and the availability of surface oxygen ligands [3,4,7]. Consequently, metal oxides represent one of the most significant classes of solid catalysts and are used for a variety of different organic reactions, including oxidation, hydrogenation, dehydrogenation, dehydration and isomerisation [3,7].

Examples of metals which have been utilised for metal oxide nanoparticles in sensors and biosensors include: zinc [8,9,10,11], copper [12,13], iron [14,15,16,17], cobalt [18,19], nickel [20,21], titanium [22,23,24] and manganese [25,26,27]. There are also many examples of mixed metal oxide (MMO) nanoparticles, consisting of oxides of two or more metals [28,29,30,31,32]. MMO nanoparticles are of interest as some examples demonstrate greater catalytic activity than their individual constituent oxides through synergistic effects [3].

There are many established methods for the synthesis of metal oxide nanoparticles such as thermal oxidation, hydrothermal, solvothermal and sol-gel methods, electrodeposition and pulsed laser deposition [33,34]. However, in recent years, so called ‘green synthesis’—wherein metal and metal oxide nanoparticles are synthesised via biological means, including bacteria, fungi, plants and plant extracts—has emerged as an interesting area of research. Green synthesis is attractive as it is simple, low-cost and environmentally friendly, avoiding the need for many environmentally damaging materials and processes [35,36].

Metal oxide nanoparticles in sensors are often used in conjunction with graphene, graphene oxide (GO) or carbon nanotubes (CNTs) [8,12,15,26]. These carbon-based nanomaterials are used both as supports for the metal oxide nanoparticles and for their advantageous characteristics, including excellent electrical properties and high surface area/volume ratios.

Propofol (2,6-diisopropylphenol) is one of the most widely used intravenous anaesthetics and is one of the most commonly used drugs in the sedation of mechanically ventilated patients in intensive care units (ICUs) [37,38,39]. The correct dosage of propofol for ICU patients is highly important, as sub-optimal sedation can result in adverse clinical outcomes and increase the time spent on ventilation [40,41,42]. However, there is a lack of reliable methods for monitoring blood propofol concentration and as many as 40–60% of patients undergoing mechanical ventilation in ICUs receive sub-optimal sedation [43]. Particular challenges for the electrochemical determination of propofol are a high degree of protein binding [44], electrode fouling that occurs when propofol is oxidised electrochemically [45] and the presence of many interfering molecules that are oxidised at similar potentials to propofol [32,46,47].

In previous publications, we have presented an enzyme-based electrochemical sensor for the continuous monitoring of propofol [48,49]. The enzyme, cytochrome P450 2B6 (CYP2B6), converts propofol into a quinone/quinol redox pair that can be easily detected without resulting in electrode fouling. In this paper, we investigate a similar CYP2B6/CNT/GO/metal oxide nanoparticle (MONP)-based electrochemical biosensor for discrete propofol measurement. By investigating both the oxidation and reduction reactions of the redox pair, we can avoid much of the likely interference, as the reduction reaction occurs at a lower potential than much of the potential interference. Various metal oxides and mixed metal oxides are investigated in order to determine the optimal nanocomposite for this application.

## 2. Materials and Methods

### 2.1. Materials

Dried bay laurel leaves were sourced from JustIngredients Ltd. (Wotton-Under-Edge, UK), and were rinsed with deionised water and dried prior to use. All other materials were purchased from Merck (Gillingham, UK) and used as supplied.

As specified by the supplier, the multi-walled carbon nanotubes (MWCNTs) have an average dimeter of 9.5 nm and length of 1.5 µm and are functionalised with carboxylic acid (>8%). The graphene oxide (GO) is 4–10% edge oxidized.

The 2,6-diisopropylphenol (97%) was made up to a 10 mM solution in dimethyl sulfoxide (DMSO, 99.9%). This solution was subsequently diluted with 10 mM phosphate buffered saline (PBS, 2.7 mM KCl, 137 mM NaCl, pH 7.4) to produce solutions with propofol concentrations ranging between 0 and 10 μg/mL as well as being used to produce spiked samples of bovine serum (adult) with propofol concentrations ranging from 0 to 10 μg/mL. These solutions were used within 24 h.

Bovine serum solutions containing paracetamol (a.k.a. acetaminophen) concentrations ranging between 0 and 25 μg/mL, L-ascorbic acid concentrations between 0 and 35 μg/mL and uric acid concentrations ranging between 0 and 70 μg/mL were also prepared in a similar manner.

### 2.2. Apparatus

A PalmSens EmStat3 potentiostat was used to perform all electrochemical measurements. The screen-printed electrodes (SPEs) consist of graphite working and counter electrodes (working electrode diameter = 1 mm) and a silver/silver chloride (Ag/AgCl) pseudo-reference electrode, and were purchased from BVT Technologies (Strážek, Czech Republic).

### 2.3. Nanocomposite Synthesis

The preparation of bay leaf extract solution has been described previously [49]. Briefly, 20 g of ground dried bay leaves were added to 200 mL of deionised water and stirred at 80 °C for 10 min. To remove any remaining plant material, the resultant solution was strained and centrifuged before being stored at 4 °C. These solutions were used within four weeks.

Cobalt (II) chloride hexahydrate, copper (II) chloride (97%), iron (III) chloride (97%), manganese (II) acetate tetrahydrate (≥99%), nickel (II) chloride hexahydrate and zinc acetate dihydrate were each dissolved in deionised water to produce 0.1 M solutions. Titanium (IV) isopropoxide was made up to a 0.1 M solution in ethanol.

Graphene oxide was suspended in 2 mL of each of the metal salt solutions at a concentration of 1 mg/mL and sonicated for 30 min to ensure maximal dispersal. A total of 100 μL of 10 g/L sodium hydroxide and 2 mL of bay leaf extraction solution was then added to each and mixed, and the mixtures were left overnight to allow for nanoparticle formation. The nanomaterial was then removed from the solution by centrifuging at 5000 rpm for 10 min and washed by re-suspending and re-centrifuging, twice in ethanol and three times in deionised water. A total of 4 mg of carbon nanotubes were then added, and the nanomaterial was suspended in deionised water to a final concentration of 0.1 mg/mL CNT and 0.05 mg/mL GO, sonicating for 2 h to ensure maximal dispersal.

For mixed metal oxide nanocomposites, the same procedure was performed using metal ion precursor solutions containing a mixture of two of the above salts with a total metal ion concentration of 0.1 M.

For the purposes of nanoparticle characterisation, an identical procedure was performed for each metal oxide, without the addition of graphene oxide or carbon nanotubes.

### 2.4. Nanoparticle Characterisation

#### 2.4.1. Ultraviolet–Visible Spectroscopy

Using a DeNovix DS-11 Fx+ spectrophotometer (Wilmington, DE, USA), UV–visible absorption spectra were recorded over the range 220–750 nm with a 10 mm path length. The metal oxide nanoparticle suspensions were sonicated for 10 min prior to measurement to ensure maximal dispersion.

#### 2.4.2. Electron Microscopy

Electron microscopy was performed using an FEI Tecnai12 BioTWIN transmission electron microscope (Hillsboro, OR, USA) fitted with a Ceta camera. The metal oxide nanoparticles were suspended in deionised water and 5 µL samples were deposited on carbon/pioloform-film-coated electron microscopy grids and left for one minute to incubate before the excess was blotted away. The samples were then imaged.

### 2.5. Electrode Functionalisation

The screen-printed electrodes were functionalised by drop-casting the CNT/GO/MONP nanocomposite solutions described in Section 2.3 using a BioDot AD1520 dispensing system. A total of 500 nL of the nanocomposite solution was deposited (in five 100 nL droplets) at the centre of the working electrode and allowed to dry at 60% relative humidity. This was then repeated twice, resulting in a total deposition of 1.5 µL of nanocomposite solution. The electrodes were then rinsed with deionised water and dried in ambient conditions.

A total of 50 μL of 10 mM PBS solution was then deposited on each electrode and cyclic voltammetry was performed between −0.6 and +0.8 V at 100 mV/s until a stable baseline was reached. The electrodes were then rinsed and dried as before.

**Figure 1:**
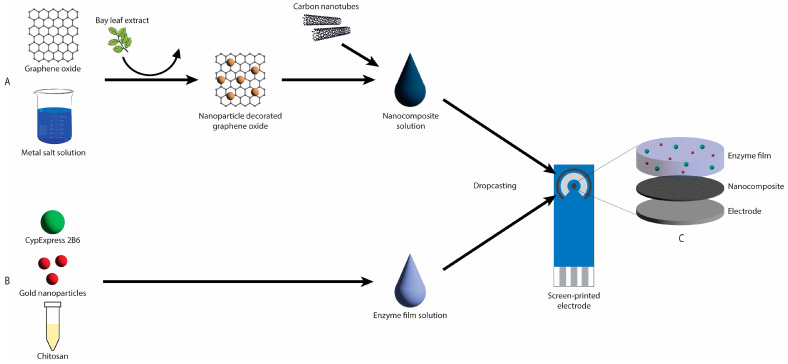
Diagrammatic representation of the electrode functionalisation protocol. (**A**) Graphene oxide is decorated with metal oxide nanoparticles via green synthesis and combined with carbon nanotubes to form the nanocomposite solution. (**B**) CypExpress 2B6, gold nanoparticles and chitosan (1%) are combined to form the enzyme film solution. (**C**) The nanocomposite and enzyme film solutions are deposited in sequence on the working electrode surface by drop-casting.

Finally, CypExpress 2B6 was suspended in 10 mM PBS (pH 7.4) at a concentration of 25 mg/mL and this suspension was mixed with a gold nanoparticle solution (approximately 0.25 mg/mL) and a 1% chitosan solution (1% acetic acid) in a ratio of 1:1:2 by volume. A total of 500 nL of this solution was drop-cast onto the working electrode using the BioDot system (5 droplets of 100 nL) and allowed to dry, and a further 500 nL was deposited on top. After drying, the electrodes were immersed in a stirred solution of 1 mM PBS for 60 min before being rinsed with deionised water and then dried in ambient conditions. The functionalised electrodes were stored at 4 °C until use and used within 48 h. The electrode functionalisation protocol is illustrated in Figure 1.

### 2.6. Electrochemical Measurement

A total of 50 μL of propofol solution (0–10 μg/mL), in either buffer or bovine serum, was deposited upon the electrode surface and differential pulse voltammetry was performed between −0.6 V and +0.8 V, with a pulse amplitude of 0.05 V, a pulse duration of 0.2 s, a step size of 0.02 V and a scan rate of 0.05 V/s (henceforth referred to as the oxidation scan). The working electrode was then held at +500 mV for a fixed period before a second differential pulse voltammetry measurement was performed between +0.5 V and −1.0 V, with a pulse amplitude of 0.05 V, a pulse duration of 0.1 s, a step size of 0.01 V and a scan rate of 0.05 V/s (henceforth referred to as the reduction scan). The electrode was then rinsed with deionised water and dried in ambient conditions.

In the case of propofol samples in bovine serum, prior to deposition upon the electrode, 45 μL of the sample was mixed with 5 μL of a 300 mM solution of ibuprofen in dimethyl sulfoxide, as described in a previous publication [50].

### 2.7. Baseline Correction

A custom MATLAB algorithm for baseline correction has been created and is described in more detail in a previous publication [50]. It performs iterative fitting of a polynomial function to the raw data to establish an appropriate baseline. In this instance, a fourth-order polynomial was used for the oxidation scan and a second-order polynomial for the reduction scan.

## 3. Results and Discussion

### 3.1. Nanoparticle Characterisation

**Figure 2:**
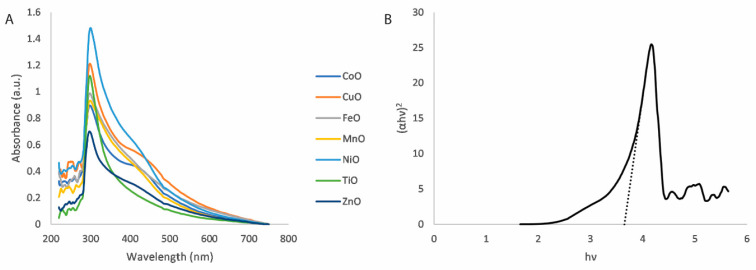
(**A**) Absorption spectra for various metal oxide nanoparticles. (**B**) Example of a Tauc plot for copper oxide nanoparticles (direct bandgap). The dashed line depicts the extrapolation of the linear absorption edge.

The absorption spectra for the various metal oxide nanoparticle dispersions are shown in Figure 2A. In each case, there is a clear absorption peak at approximately 300 nm. The bandgap energies of the nanoparticles can be estimated from these absorption spectra using the Tauc method [51,52]. The optical absorption strength is provided by the following:

(1) $${\left(\alpha h\nu \right)}^{1/n}=A(h\nu -E_{g})$$

where α is the absorption coefficient, h is Plank’s constant, ν is the optical frequency, A is a constant of proportionality and $E_{g}$ is the bandgap energy. The value of n denotes the manner of the electron transition. In the Tauc method, $E_{g}$ is determined by the intercept of the extrapolation of the linear absorption edge with the *x*-axis. An example of this is shown in Figure 2B for the direct bandgap (n=1/2); equivalent plots for the other metal oxide and mixed metal oxide nanoparticles can be found in the Supplementary Information (Figures S1–S12).

The bandgap energies determined in this manner for each of the metal oxide nanoparticles are shown in Table 1. They range from approximately 3.3 to 3.8 eV and the relative similarity across all the metal oxides suggests that this method of nanoparticle synthesis produces nanoparticles of similar physical dimensions irrespective of the metal, as it is known that quantum size effects greatly influence the optical properties of nanomaterials [53]. The equivalent bandgap energies for the mixed metal oxide nanoparticles are presented in the Supplementary Information, Table S1.

**Figure 3:**
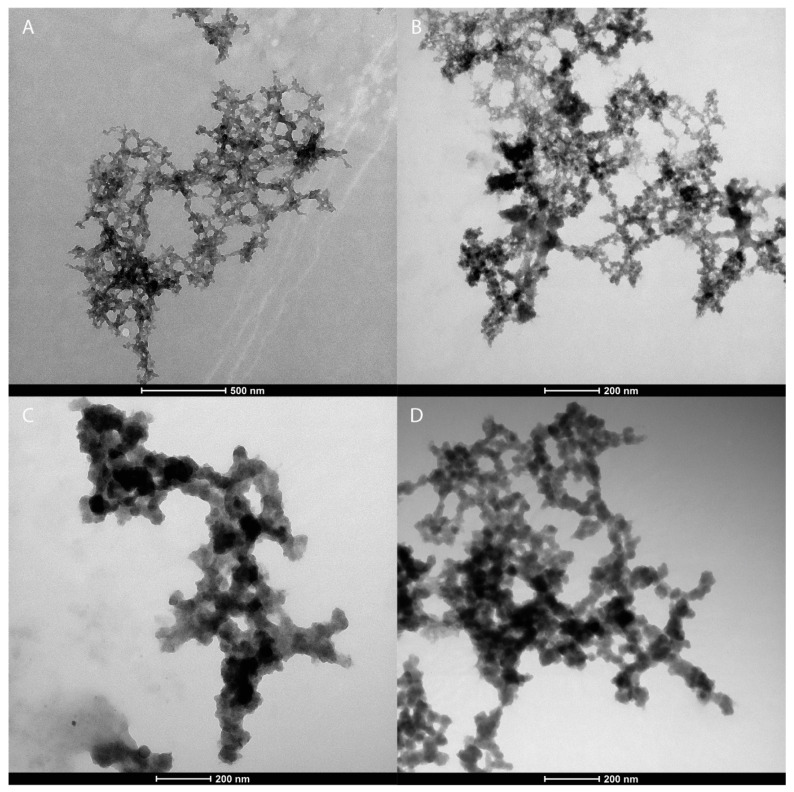
Transmission electron microscopy images of (**A**) cobalt oxide nanoparticles, (**B**) titanium oxide nanoparticles, (**C**) copper titanium oxide nanoparticles, and (**D**) zinc titanium oxide nanoparticles.

The successful synthesis of the metal oxide nanoparticles is confirmed via transmission electron microscopy, examples of which are shown in Figure 3. The micrographs show that the metal oxide nanoparticles are approximately spheroid in shape and have sizes ranging between approximately 5 and 50 nm.

### 3.2. Electrochemical Measurement

#### 3.2.1. Dual Scan Optimisation

The dual scan measurement procedure described in Section 2.6 was carried out for functionalised electrodes with varying times at which the electrodes were held at +500 mV (amperometry times). It was found that the greatest sensitivity for the reduction scan was produced with an amperometry time of 2 min (see Supplementary Information Figure S13), and this time was used for all subsequent measurements.

#### 3.2.2. Metal Oxide Nanocomposites

As discussed in a previous publication [49], CNT and GO are selected as a means of enhancing the performance of the sensor based on their high surface area/volume ratios and their excellent electrochemical characteristics. The GO is used as scaffold for the metal oxide and mixed metal oxide nanoparticles, and the CNTs are added as spacers to prevent agglomeration and improve the dispersion of the nanocomposite material.

An example of the differential pulse voltammograms for an electrode functionalised with a CNT/GO/CoONP nanocomposite for solutions of varying propofol concentration are shown in Figure 4. On the oxidation scan, the concentration-dependent oxidation peak of 2,6-diisopropyl quinol can be clearly seen at approximately 400 mV (Figure 4A), whereas for the reduction scan the concentration-dependent reduction peak of 2,6-diisopropyl quinone can be clearly seen at approximately −100 mV (Figure 4B). The peak current values versus the propofol concertation for each scan are shown in Figure 5. In both cases, the sensor produces a linear response across the therapeutic range of propofol (1–10 µg/mL [54]). Equivalent plots for each of the different metal oxides can be found in the Supplementary Information (Figures S14–S37).

**Figure 6:**
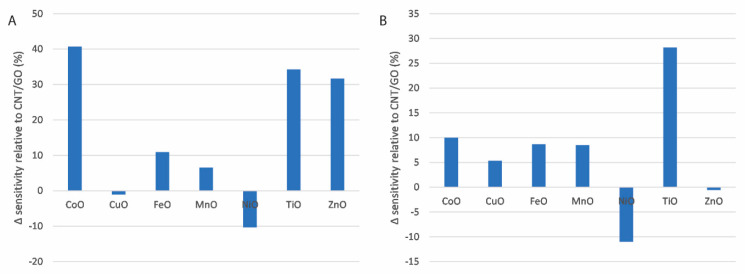
Change in sensitivities for each electrode as a percentage relative to CNT/GO for each metal oxide for (**A**) oxidation scan and (**B**) reduction scan.

The sensitivities for sensors prepared with each of the CNT/GO/MONP nanocomposites are shown, relative to the equivalent for electrodes functionalised with only a mixture of carbon nanotubes and graphene oxide, in Figure 6. The sensitivities for the oxidation reactions are shown in Figure 6A and the equivalent values for the reduction reaction are shown in Figure 6B. Nanomaterials can enhance sensor performance by increasing the surface area of the electrode, improving electron transfer and catalytic effects. Any improvements to surface area or electron transfer are likely to be modest when compared to that of the carbon nanotube/graphene oxide mixture [49], so displaying the sensitivities relative to a CNT/GO electrode is an effective way of isolating the catalytic effects of the metal oxide nanoparticles. It can be seen from Figure 6A that, for the oxidation scan, cobalt oxide nanoparticles impart the greatest improvement in sensitivity with an approximately 40% increase compared to CNT/GO, followed by titanium oxide and zinc oxide at approximately 34% and 32%, respectively. Iron oxide and manganese oxide impart a modest improvement, and copper oxide and nickel oxide result in a decrease in sensitivity, most likely as a result of a combination of low catalytic activity, coupled with a reduction in the total effective area of available graphene oxide. In the case of the reduction scan, it can be seen from Figure 6B that titanium oxide nanoparticles impart the greatest improvement in sensitivity at approximately 28%, compared to approximately 10% or lower for the oxides of cobalt, copper, iron and manganese and decreases in sensitivity for nickel oxide and zinc oxide. From this, it can be concluded that, of the single metal oxides investigated, titanium oxide is the most promising for this dual scan technique.

#### 3.2.3. Mixed Metal Oxide Nanocomposites

**Figure 7:**
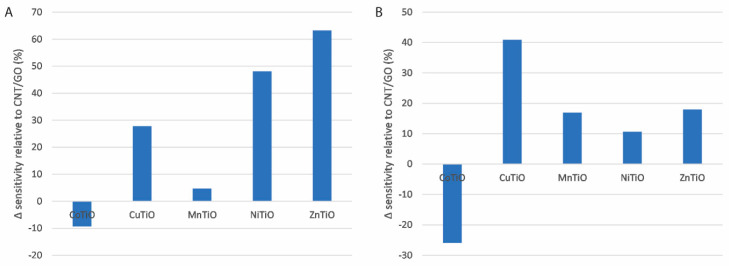
Change in sensitivities for each electrode as a percentage relative to CNT/GO for each MMO for the (**A**) oxidation scan and (**B**) reduction scan.

MMO nanocomposites were prepared as described in Section 2.3 using precursor solutions of metal ion mixtures with a total metal ion concentration of 100 mM, consisting of two parts Ti^4+^ to one part M, where M is a metal ion other than titanium. The sensitivities for sensors prepared with each of these CNT/GO/MMONP nanocomposites are shown, relative to the equivalent for electrodes functionalised with only a mixture of carbon nanotubes and graphene oxide, in Figure 7. The sensitivities for the oxidation reactions are shown in Figure 7A and the equivalent values for the reduction reaction are shown in Figure 7B. From Figure 7A it can be seen that for the oxidation scan, only composites containing nickel titanium oxide or zinc titanium oxide offer greater improvements in sensitivity than titanium oxide nanoparticles, at approximately 48% and 63%, respectively. In the case of the reduction scan, Figure 7B shows that only nanocomposites containing copper titanium oxide nanoparticles offer an improvement in sensitivity greater than titanium oxide alone, at approximately 41% compared to CNT/GO. The fact that metals such as copper and nickel, which showed poor catalytic performance as single metal oxides, show such improved performance as part of mixed metal oxides alongside titanium is evidence of the synergistic effects of mixed metal oxides discussed previously. This is potentially due to the formation of p/n heterojunctions within the nanomaterial [55,56,57].

The exact mechanism or mechanisms that determine which metal oxides or mixed metal oxides display superior catalytic performance for the reactions in question are not clear. While the formation of heterojunctions may play a role, there are many other factors that may contribute. This will be an interesting area for future study.

It can be concluded that the optimum nanocomposite depends on which scan is of greater interest. Nanocomposites incorporating zinc titanium oxide nanoparticles will offer the greatest sensitivity for the oxidation scan, whereas nanocomposites incorporating copper titanium oxide will offer the greatest sensitivity for the reduction scan.

Nanocomposites containing iron titanium oxide nanoparticles were prepared, but for reasons that are unclear, these nanocomposites did not disperse well in water, falling out of suspension rapidly, and were discounted for this reason.

**Figure 8:**
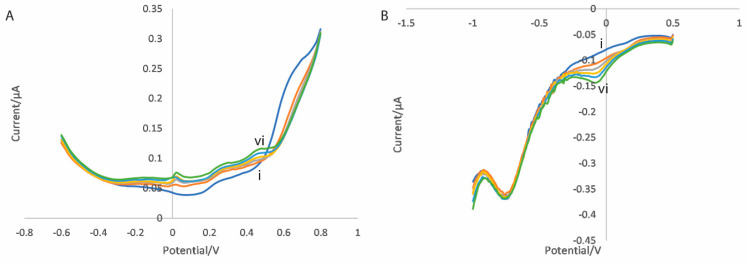
Differential pulse voltammograms for the (**A**) oxidation scan and (**B**) reduction scan for a CNT/GO/CuTiONP-nanocomposite-functionalised electrode in bovine serum with propofol concentrations of (i) 0, (ii) 2, (iii) 4, (iv) 6, (v) 8 and (vi) 10 µg/mL.

Electrodes functionalised with CNT/GO/CuTiONP and CNT/GO/ZnTiONP nanocomposites were used to perform the dual scan measurement procedure described above for propofol-spiked bovine serum solutions. In order to overcome the issue of the very high protein binding of propofol, a molecular displacement solution of ibuprofen was used as described in a previous publication [50]. Examples of differential pulse voltammograms produced in these measurements for a CNT/GO/CuTiONP electrode are shown in Figure 8 (with the equivalent for a CNT/GO/ZnTiONP electrode shown in Supplementary Information Figure S38). Concentration-dependent oxidation and reduction peaks are still evident at approximately +460 and −60 mV, respectively, although with reduced amplitudes as a result of protein binding effects.

**Figure 9:**
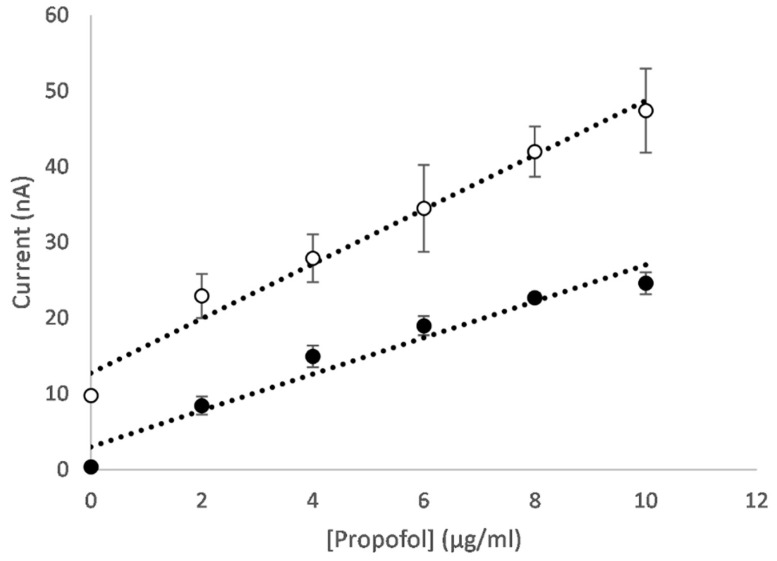
Peak current versus propofol concentration at +460 mV for the oxidation scan (black circles) and −60 mV for the reduction scan (white circles) for CNT/GO/CuTiO electrodes in spiked bovine serum. Baseline correction has been applied and the error bars represent one standard deviation (N = 3).

The peak current versus propofol concentration for both the oxidation and reduction scans of CNT/GO/CuTiONP-functionalised electrodes are shown in Figure 9. Interestingly, whereas in buffer solution the sensitivity produced from the oxidation scan appears greater than that from the reduction scan (presumably as a result of the enzyme favouring the production of the quinol over the quinone) irrespective of the nanomaterial used, in serum, the opposite appears to be the case. This is most likely to be a result of protein binding effects, with the quinone having a lower binding affinity to albumin than the quinol, leading to a larger free fraction.

The sensitivities and detection limits (calculated by $3\;\sigma_{0}/gradient$) for both scans for both CNT/GO/CuTiONP- and CNT/GO/ZnTiONP-functionalised electrodes are shown in Table 2. As stated previously, the reduction scans display significantly higher sensitivities, at 4.58 and 4.34 nA/μg/mL/mm^2^ for CuTiO and ZnTiO, respectively, compared to 3.05 and 3.03 nA/μg/mL/mm^2^, respectively, for the oxidation scans. The significant differences in sensitivity between the two mixed metal oxides evident in the measurements in buffer are not evident in the serum measurements. This is likely a result of protein binding, as the effective analyte concentration will be much reduced in serum, rendering any differences between the nanocomposites less pronounced. However, nanocomposites incorporating CuTiONP do demonstrate significantly lower detection limits, 0.46 and 0.47 μg/mL for the oxidation and reduction scans, respectively, compared to those for nanocomposites incorporating ZnTiONP, at 3.33 and 6.15 μg/mL, respectively, as a result of significantly lower variation between electrodes. This suggests that CNT/GO/CuTiONP nanocomposites are a better choice for electrochemical propofol sensors.

This detection limit of 0.5 μg/mL is below the lower limit of the therapeutic range for propofol (1–10 μg/mL [54]) and is the same order of magnitude as reported for optical detection methods, such as the fluorescence-based methods of Šrámková et al. [58] and Diao et al. [59] (1.3 and 0.5 μg/mL, respectively, in propofol emulsions) and the spectrophotometry-based approaches of Liu et al. [60] and Gad-Kariem and Abounassif [61] (0.25 μg/mL in whole blood and 0.28 μg/mL in plasma, respectively). It is also of the same order of magnitude as that obtained via a chemiresistive molecularly imprinted polymer, as reported by Hong et al. [62] (0.1 μg/mL in plasma).

This sensitivity of 4.58 nA/μg/mL/mm^2^ is a considerable improvement upon that which we reported in a previous publication [50] using differential pulse voltammetry and a CNT/GO/FeONP-nanocomposite-functionalised electrode. Therein we reported a sensitivity of 2.82 nA/μg/mL/mm^2^ in bovine serum, meaning that this CNT/GO/CuTiONP nanocomposite in conjunction with the dual scan methodology described herein offers an improvement to the sensitivity of greater than 60%.

#### 3.2.4. Interference

A significant challenge in developing any electrochemical sensor for analytes in blood or serum is the presence of other electroactive molecules that can present potential sources of interference. For propofol monitoring, perhaps the most significant such molecule is the analgesic drug paracetamol (also known as acetaminophen). It is widely used in both domestic and clinical settings [63,64], with one study reporting that 64% of ICU patients received paracetamol as part of their treatment [65]. It is oxidised at similar potentials to propofol [66], has a similar therapeutic range [54] and has a lower protein binding affinity [44,67,68], resulting in higher free fractions.

**Figure 10:**
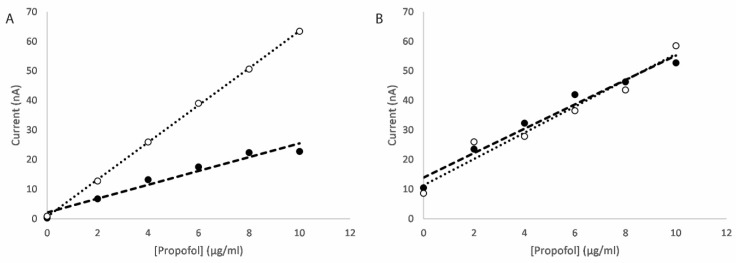
Peak current against propofol concentration for (**A**) the oxidation scan (at +460 mV) and (**B**) the reduction scan (at −60 mV) for bovine serum samples spiked with propofol (black circles) and with a mixture of propofol and paracetamol (white circles). The paracetamol concentration is 2.5 times that of the propofol concentration.

In order to investigate the selectivity of these sensors towards paracetamol, the measurement procedure described previously was performed using electrodes functionalised with the CNT/GO/CuTiONP nanocomposite and bovine serum samples containing a mixture of both propofol and paracetamol. The propofol concentration was varied between 0 and 10 μg/mL, as previously, with the paracetamol concentration fixed at 2.5 times the propofol concentration (resulting in a concentration range between 0 and 25 μg/mL) to account for the differences in their respective therapeutic ranges. An example of the peak current against propofol concentration for both the oxidation and reduction scans is shown in Figure 10, plotted alongside equivalent data from an identical electrode in serum solutions containing only propofol.

It can be seen that, for the oxidation scan (Figure 10A), the currents produced at +460 mV in solutions containing both propofol and paracetamol are significantly larger than those for solutions containing propofol alone, and that this difference is proportional to the paracetamol concentration, indicating significant interference as a result of the presence of paracetamol. However, it can be seen from Figure 10B that the currents at −60 mV in the reduction scan are very similar for both serum solutions containing a combination of propofol and paracetamol and propofol alone, and display no apparent dependence on paracetamol concentration. This shows that paracetamol is not a source of interference at the lower potential of the reduction scan.

**Figure 11:**
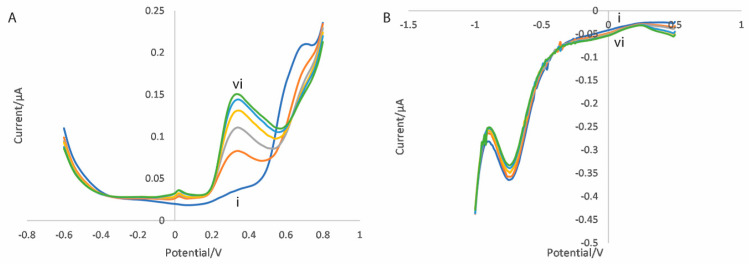
Differential pulse voltammograms for (**A**) the oxidation scan and (**B**) the reduction scan for uric acid in bovine serum at concentrations of (i) 0, (ii) 14, (iii) 28, (iv) 42, (v) 56 and (vi) 70 µg/mL.

An additional significant potential source of interference for electrochemical sensors operating in biological fluids is uric acid, which is electroactive and present in blood at significant concentrations [46,69,70]. Figure 11 shows differential pulse voltammograms for both the oxidation and reduction scans for bovine serum samples spiked with uric acid concentrations ranging from 0 and 70 μg/mL (covering the expected range in human blood [71]). For the oxidation scan (Figure 11A), there is a significant concentration-dependent peak at approximately +350 mV which overlaps with the relevant potential for propofol and the associated quinol (approximately +460 mV), which would result in significant interference. However, in the reduction scan (Figure 11B), any variation caused by the presence of uric acid at the potential of interest for the propofol-associated quinone (approximately −60 mV) is negligible. This shows that, like paracetamol, at the higher potentials of the oxidation scan, uric acid is a significant potential source of interference, but that at the lower potentials of the reduction scan, uric acid does not present a significant potential source of interference. Similar results were obtained for ascorbic acid, another potential source of interference [46,72,73] (Supplementary Information, Figure S40).

## 4. Concluding Remarks

We have presented a cytochrome P450 2B6/carbon nanotube/graphene oxide/metal oxide nanoparticle nanocomposite-based electrochemical sensor for the discrete measurement of the intravenous anaesthetic propofol. The enzyme converts propofol into a quinol/quinone redox pair, allowing for measurement over two distinct potential ranges. The nanocomposite allows for sensitive detection, with the metal oxide nanoparticles contributing a catalytic effect. We have investigated various metal oxide and mixed metal oxide nanoparticles as potential nanocatalysts and found that, of those tested, nanocomposites containing copper titanium oxide produce the best overall performance.

The metal oxide nanoparticles were synthesised via green synthesis, making this paper one of few to investigate this approach for sensor development, and one of very few to investigate green synthesis for the formation of mixed metal oxide nanoparticles [36,74,75]. This approach offers a faster, lower-cost and more sustainable methodology for nanomaterial synthesis for sensor applications. However, more investigations are required in order to provide a direct comparison with conventional synthesis approaches in terms of parameters such as particle size, polydispersity and reproducibility, as well as the potential for scaling this approach.

CNT/GO/CuTiONP-functionalised electrodes demonstrate a sensitivity of 4.58 nA/μg/mL/mm^2^ in bovine serum, representing a significant improvement compared to our previously reported CNT/GO/FeONP-based sensor. The corresponding detection limit is 0.5 μg/mL, comfortably below the lower end of the therapeutic range for propofol.

We have shown that by measuring at the lower potential range (corresponding to the reduction of 2,6-diisopropyl quinone), it is possible to avoid the potential interference caused by paracetamol, uric acid and ascorbic acid, rendering this technique highly specific.

The principal obstacle to achieving a lower limit of detection is the device-to-device variability. Future work will investigate methods of reducing this, such as the incorporation of an internal standard for self-calibration.

## Figures and Tables

**Figure 4:**
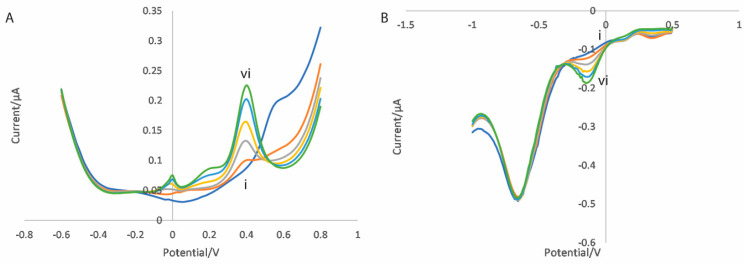
Differential pulse voltammograms for (**A**) the oxidation scan and (**B**) the reduction scan of a CNT/GO/CoONP nanocomposite-functionalised electrode in 10 mM PBS with propofol concentrations of (i) 0, (ii) 2, (iii) 4, (iv) 6, (v) 8 and (vi) 10 µg/mL.

**Figure 5:**
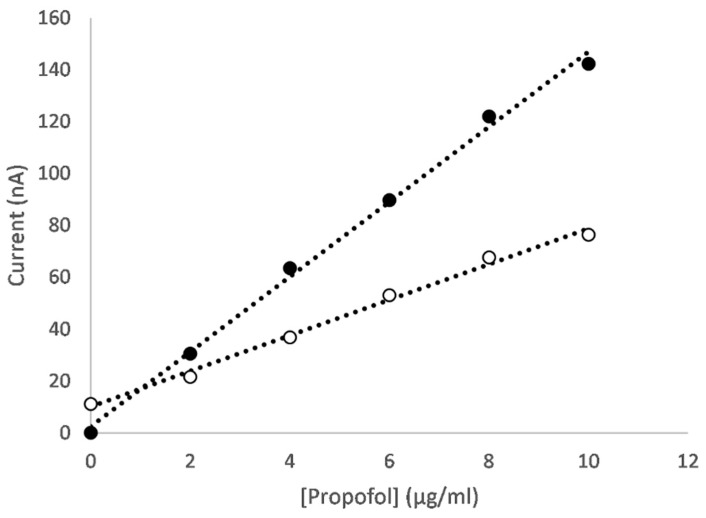
Peak current versus propofol concentration at +400 mV for the oxidation scan (black circles) and −110 mV for the reduction scan (white circles) for a CNT/GO/CoONP nanocomposite-functionalised electrode. Baseline correction has been applied.

**Table 1:** Bandgap energies for the various metal oxide nanoparticles for the direct bandgap, as determined by the Tauc method.

Metal Oxide Nanoparticle	Bandgap Energy (eV)
Cobalt oxide	3.56
Copper oxide	3.65
Iron oxide	3.46
Manganese oxide	3.39
Nickel oxide	3.56
Titanium oxide	3.73
Zinc oxide	3.75

**Table 2:** Sensitivities and detection limits for first and second scans for electrodes functionalised with nanocomposites containing CuTiO and ZnTiO nanoparticles.

Scan	Nanocatalyst	Sensitivity (nA/μg/mL/mm^2^)	Detection Limit (μg/mL)
Oxidation	CuTiO	3.05	0.46
Oxidation	ZnTiO	3.03	3.33
Reduction	CuTiO	4.58	0.47
Reduction	ZnTiO	4.34	6.15

## Data Availability

The raw data supporting the conclusions of this article will be made available by the authors on request.

## Supplementary Materials

The following supporting information can be downloaded at: https://www.mdpi.com/article/10.3390/mi16020120/s1, Figure S1: UV-vis spectrum for CoO nanoparticles; Figure S2: UV-vis spectrum for CuO nanoparticles; Figure S3: UV-vis spectrum for FeO nanoparticles; Figure S4: UV-vis spectrum for MnO nanoparticles; Figure S5: UV-vis spectrum for NiO nanoparticles; Figure S6: UV-vis spectrum for TiO nanoparticles; Figure S7: UV-vis spectrum for ZnO nanoparticles; Figure S8: UV-vis spectrum for CoTiO nanoparticles; Figure S9: UV-vis spectrum for CuTiO nanoparticles; Figure S10: UV-vis spectrum for MnTiO nanoparticles; Figure S11: UV-vis spectrum for NiTiO nanoparticles; Figure S12: UV-vis spectrum for ZnTiO nanoparticles; Table S1: Band gap energies for the various metal oxide nanoparticles for the direct band-gap, as determined by the Tauc method; Figure S13: Current vs. propofol concentration for CNT/GO/FeONP functionalised electrodes with varying amperometry times; Figure S14: Differential pulse voltammetry for CoO electrode; Figure S15: Current versus propofol concentration for CoO electrode; Figure S16: Differential pulse voltammetry for CuO electrode; Figure S17: Current versus propofol concentration for CuO electrode; Figure S18: Differential pulse voltammetry for FeO electrode; Figure S19: Current versus propofol concentration for FeO electrode; Figure S20: Differential pulse voltammetry for MnO electrode; Figure S21: Current versus propofol concentration for MnO electrode; Figure S22: Differential pulse voltammetry for NiO electrode; Figure S23: Current versus propofol concentration for NiO electrode; Figure S24: Differential pulse voltammetry for TiO electrode; Figure S25: Current versus propofol concentration for TiO electrode; Figure S26: Differential pulse voltammetry for ZnO electrode; Figure S27: Current versus propofol concentration for ZnO electrode; Figure S28: Differential pulse voltammetry for CoTiO electrode; Figure S29: Current versus propofol concentration for CoTiO electrode; Figure S30: Differential pulse voltammetry for CuTiO electrode; Figure S31: Current versus propofol concentration for CuTiO electrode; Figure S32: Differential pulse voltammetry for MnTiO electrode; Figure S33: Current versus propofol concentration for MnTiO electrode; Figure S34: Differential pulse voltammetry for NiTiO electrode; Figure S35: Current versus propofol concentration for NiTiO electrode; Figure S36: Differential pulse voltammetry for ZnTiO electrode; Figure S37: Current versus propofol concentration for ZnTiO electrode; Figure S38: Differential pulse voltammograms for CNT/GO/ZnTiONP nanocomposite electrode in bovine serum; Figure S39: Peak current versus propofol current for oxidation and reduction scans in bovine serum for CNT/GO/ZnTiONP electrode; Figure S40: Differential pulse voltammetry scans for ascorbic acid in bovine serum.
