# Supplementary material for: Metal Oxide Nanocatalysts for the Electrochemical Detection of Propofol
Source: Micromachines (Basel). 2025 Jan 22;16(2):120. doi: 10.3390/mi16020120 (PMC11857172; doi:10.3390/mi16020120)
Supplement: Supplementary file 1 [file micromachines-16-00120-s001.zip › micromachines-3369491-supplementary.pdf]

Metal oxide nano-catalysts for electrochemical detection of propofol: Supplementary information

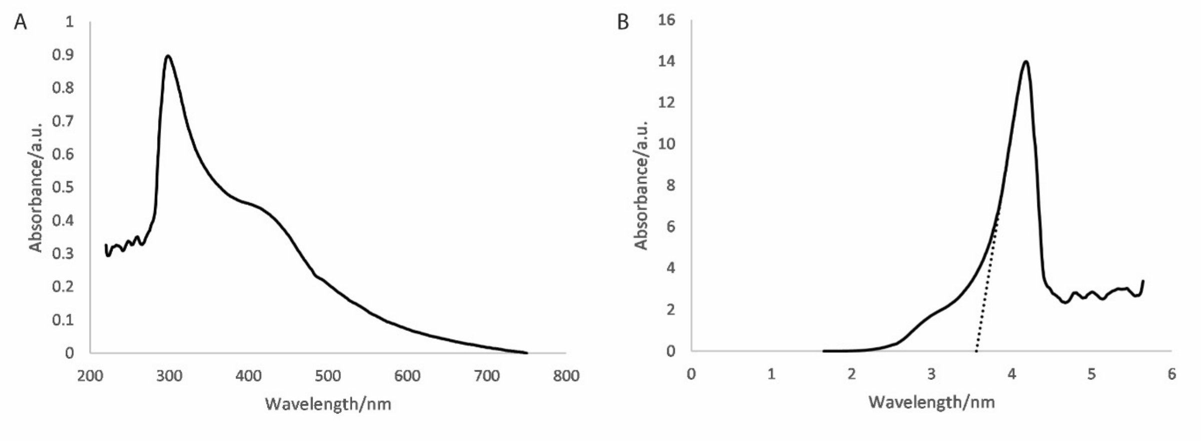

Figure S1 – A) UV-vis spectrum for CoO nanoparticles. B) Corresponding Tauc plot, the dashed line represents the extrapolation of the linear absorption edge.

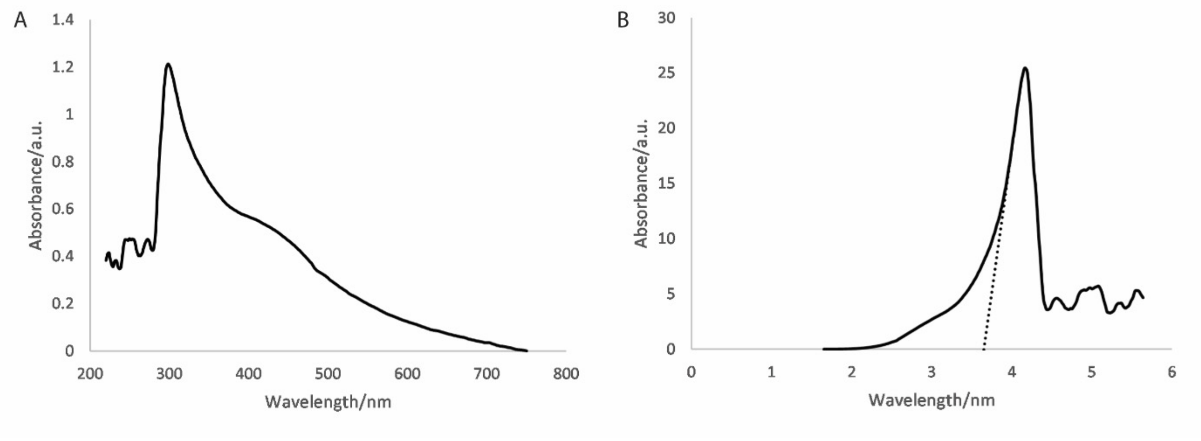

Figure S2 – A) UV-vis spectrum for CuO nanoparticles. B) Corresponding Tauc plot, the dashed line represents the extrapolation of the linear absorption edge.

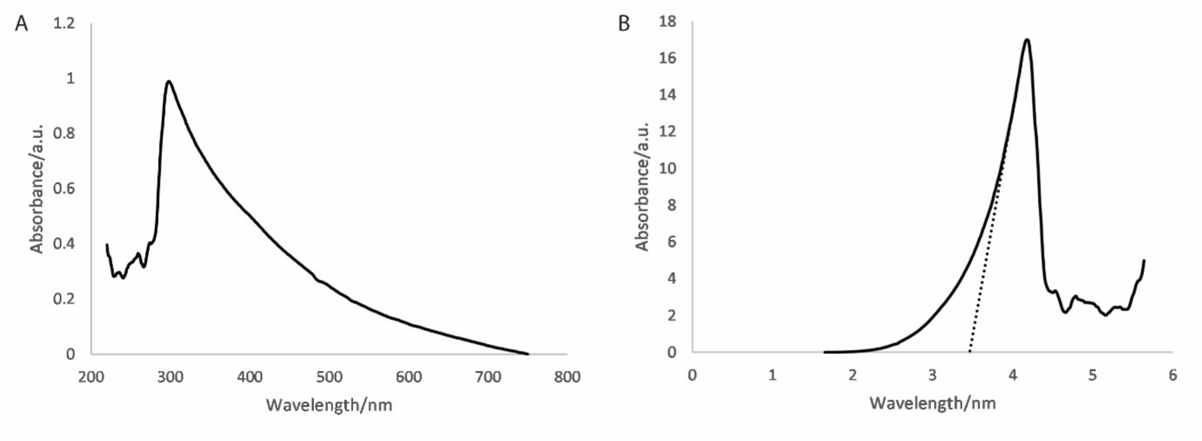

Figure S3 – A) UV-vis spectrum for FeO nanoparticles. B) Corresponding Tauc plot, the dashed line represents the extrapolation of the linear absorption edge.

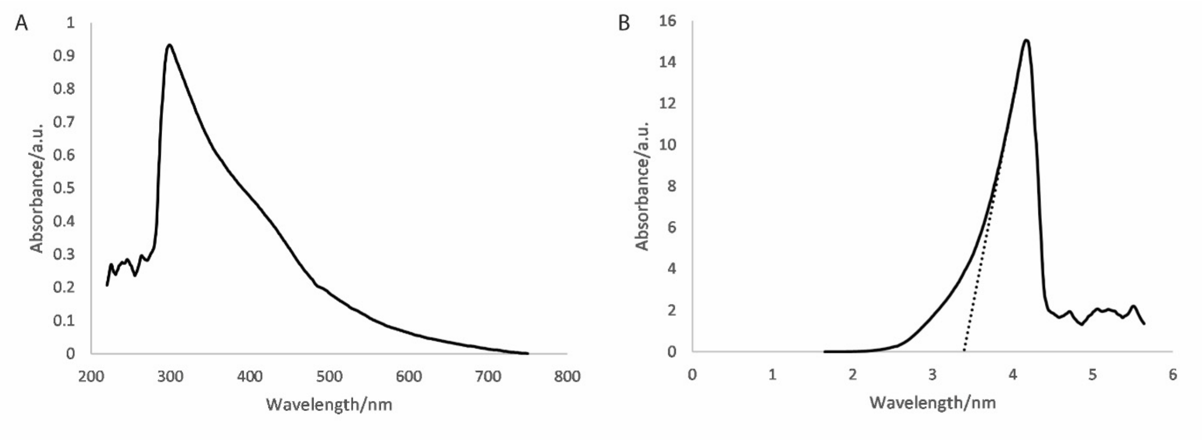

Figure S4 – A) UV-vis spectrum for MnO nanoparticles. B) Corresponding Tauc plot, the dashed line represents the extrapolation of the linear absorption edge.

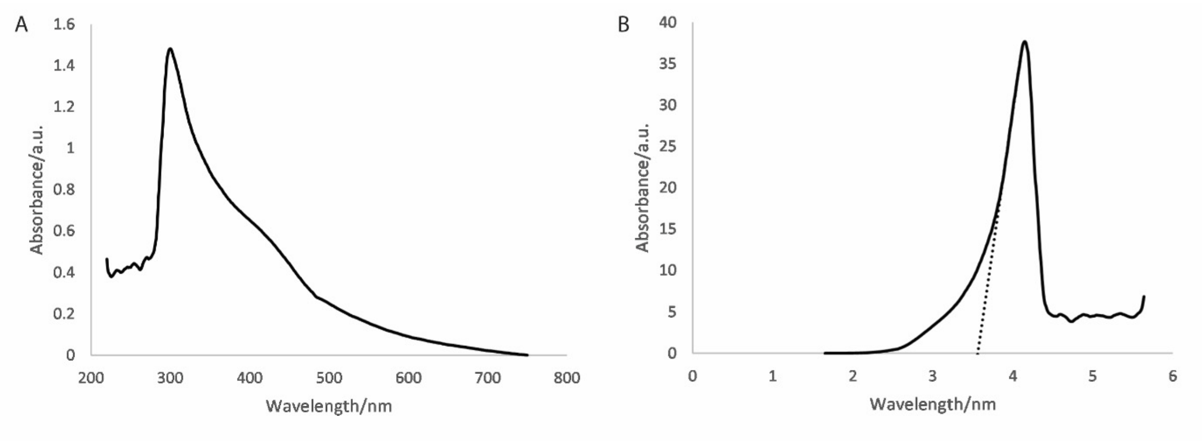

Figure S5 – A) UV-vis spectrum for NiO nanoparticles. B) Corresponding Tauc plot, the dashed line represents the extrapolation of the linear absorption edge.

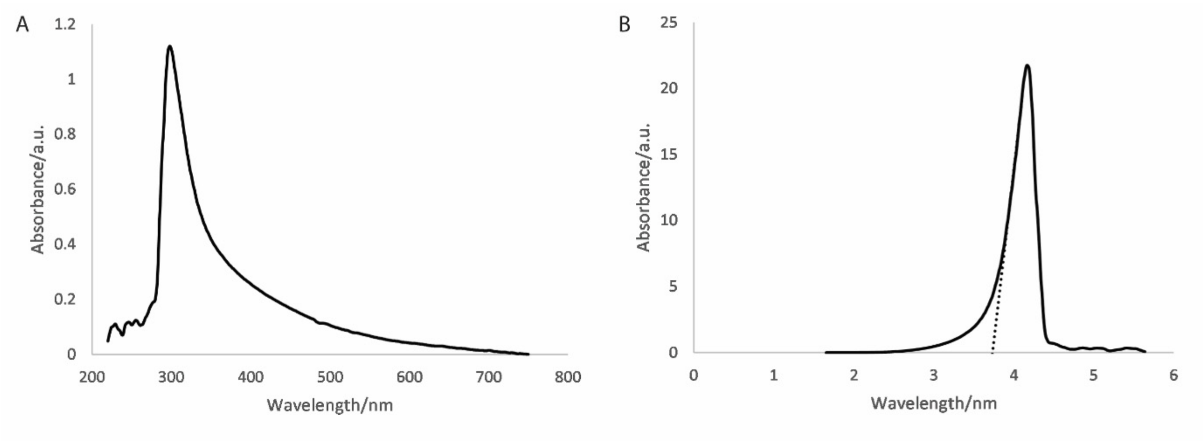

Figure S6 – A) UV-vis spectrum for TiO nanoparticles. B) Corresponding Tauc plot, the dashed line represents the extrapolation of the linear absorption edge.

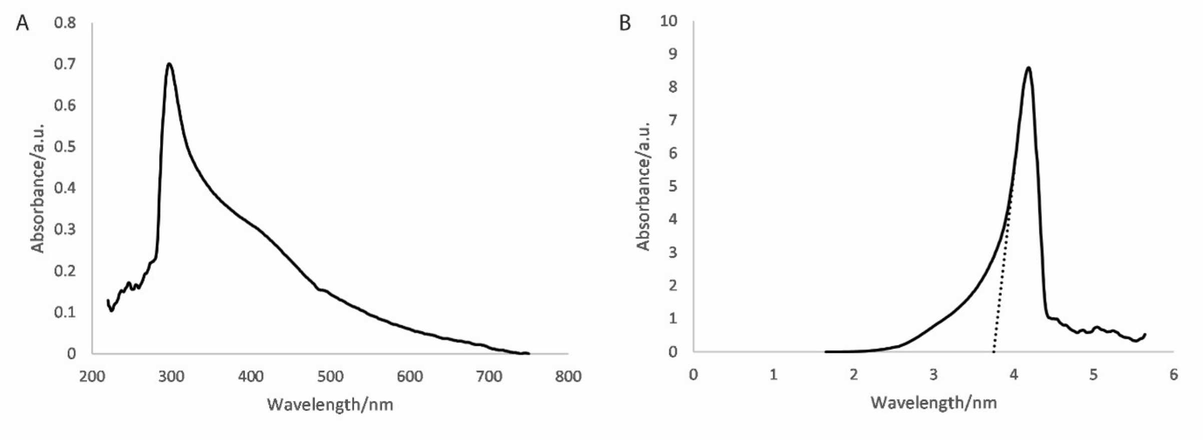

Figure S7 – A) UV-vis spectrum for ZnO nanoparticles. B) Corresponding Tauc plot, the dashed line represents the extrapolation of the linear absorption edge.

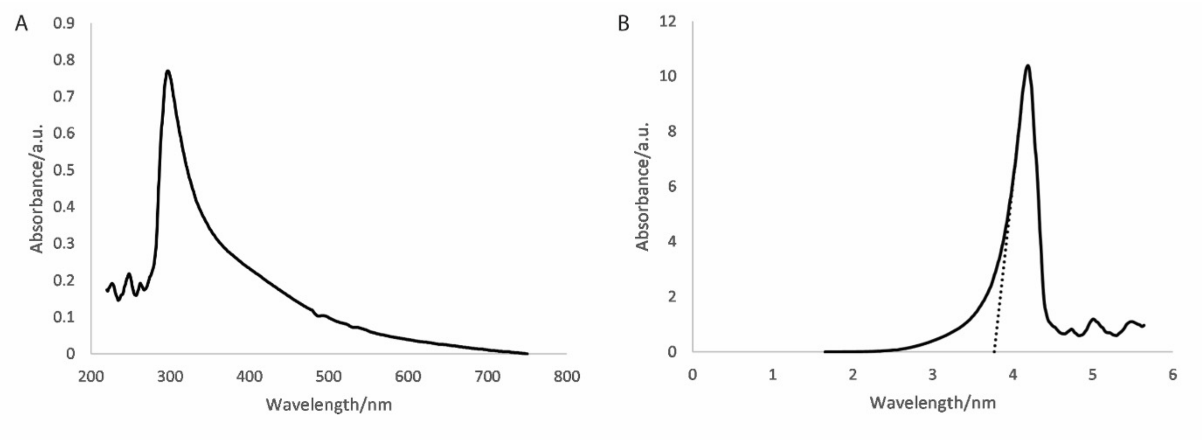

Figure S8 – A) UV-vis spectrum for CoTiO nanoparticles. B) Corresponding Tauc plot, the dashed line represents the extrapolation of the linear absorption edge.

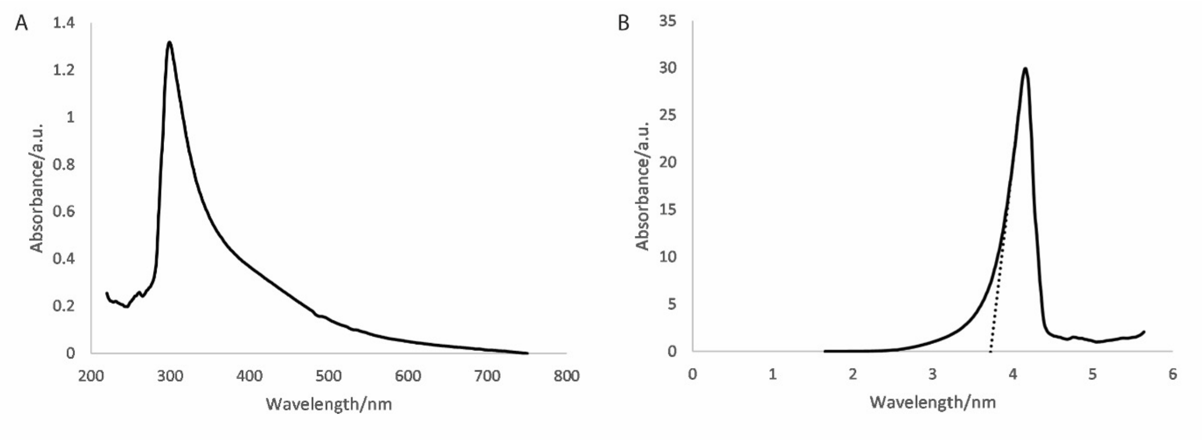

Figure S9 – A) UV-vis spectrum for CuTiO nanoparticles. B) Corresponding Tauc plot, the dashed line represents the extrapolation of the linear absorption edge.

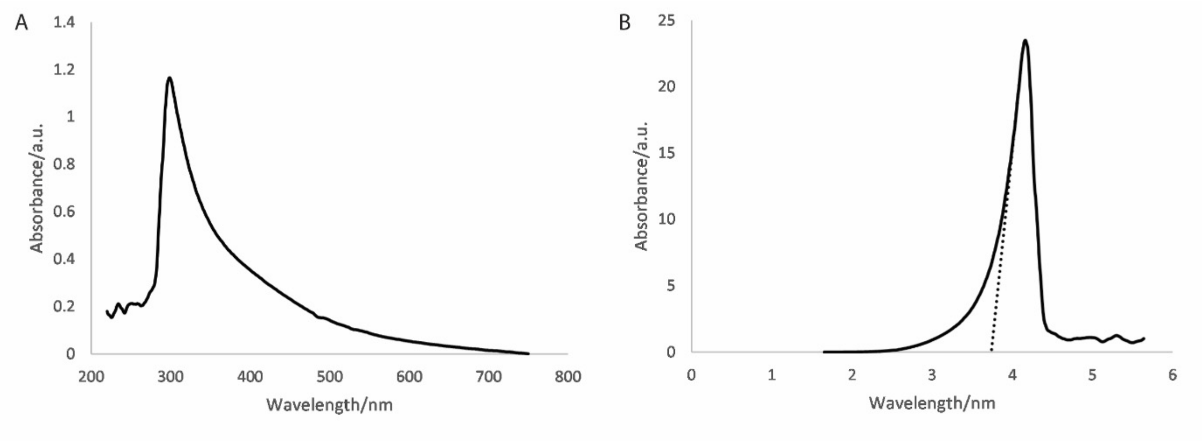

Figure S10 – A) UV-vis spectrum for MnTiO nanoparticles. B) Corresponding Tauc plot, the dashed line represents the extrapolation of the linear absorption edge.

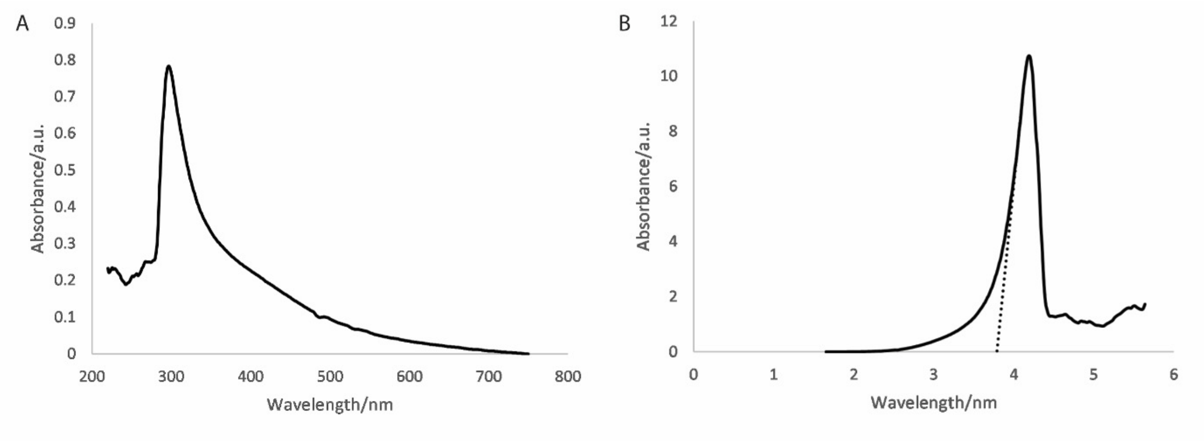

Figure S11 – A) UV-vis spectrum for NiTiO nanoparticles. B) Corresponding Tauc plot, the dashed line represents the extrapolation of the linear absorption edge.

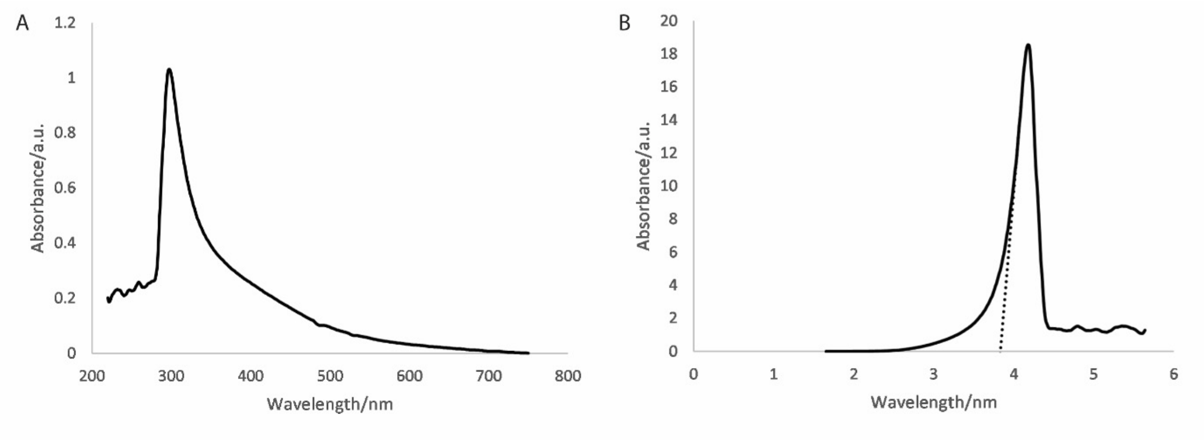

Figure S12 – A) UV-vis spectrum for ZnTiO nanoparticles. B) Corresponding Tauc plot, the dashed line represents the extrapolation of the linear absorption edge.

**Mixed metal oxide nanoparticle**	**Band-gap energy (eV)**
Cobalt titanium oxide	3.77
Copper titanium oxide	3.72
Manganese titanium oxide	3.74
Nickel titanium oxide	3.79
Zinc titanium oxide	3.83

Table S1 – Band gap energies for the various metal oxide nanoparticles for the direct band-gap, as determined by the Tauc method.

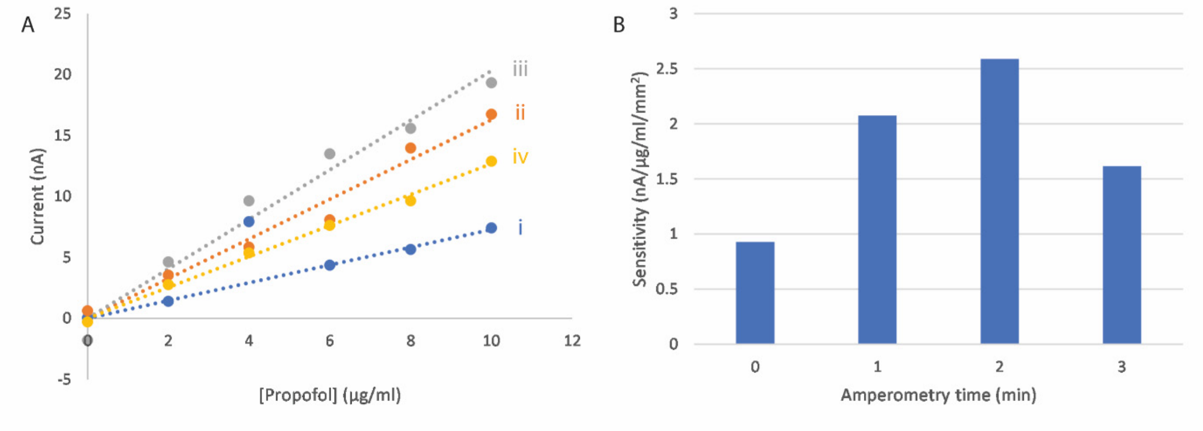

Figure S13 – A) Current vs. propofol concentration for CNT/GO/FeONP functionalised electrodes with amperometry times of i) 0, ii) 1, iii) 2 and iv) 3 minutes. B) Sensitivity obtained for each different amperometry time.

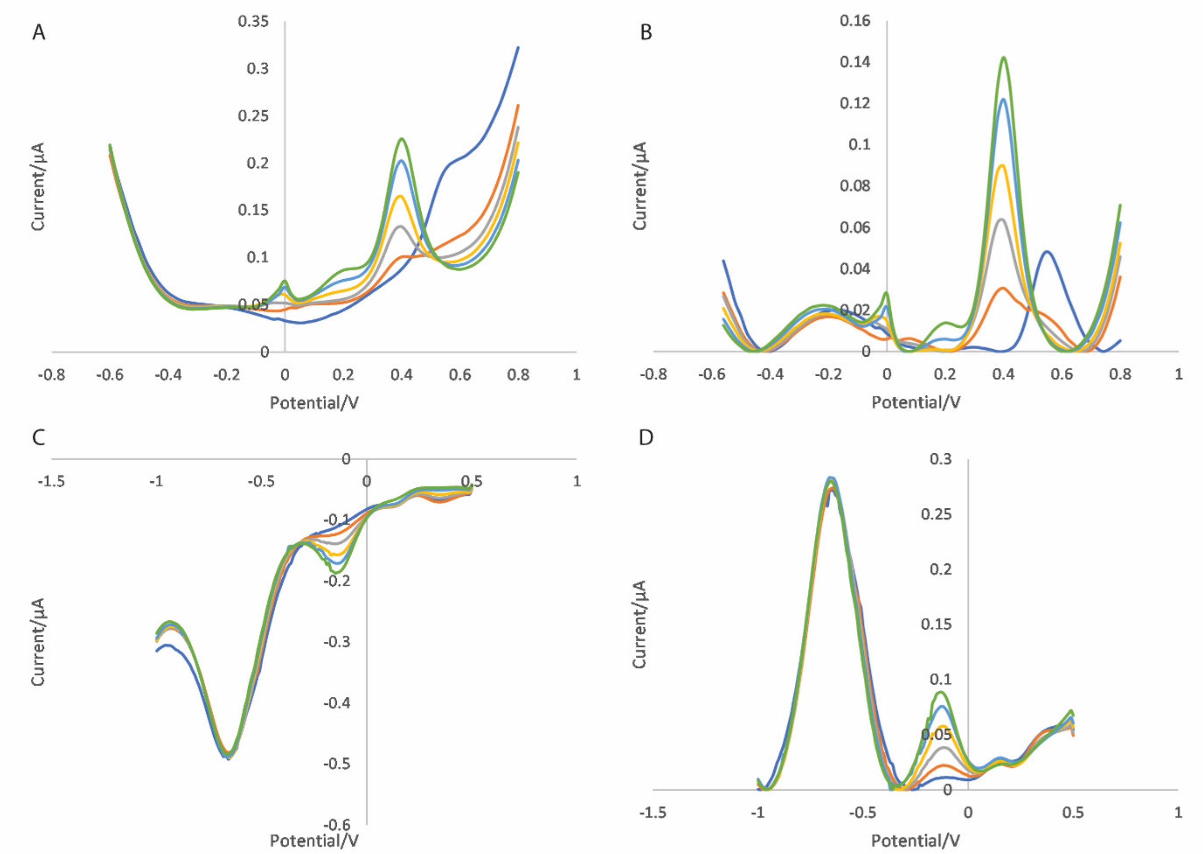

Figure S14 – Differential pulse voltammetry for CoO electrode: A) Scan 1 - raw, B) Scan 1 - baseline corrected, C) Scan 2 - raw, D) Scan 2 - baseline corrected.

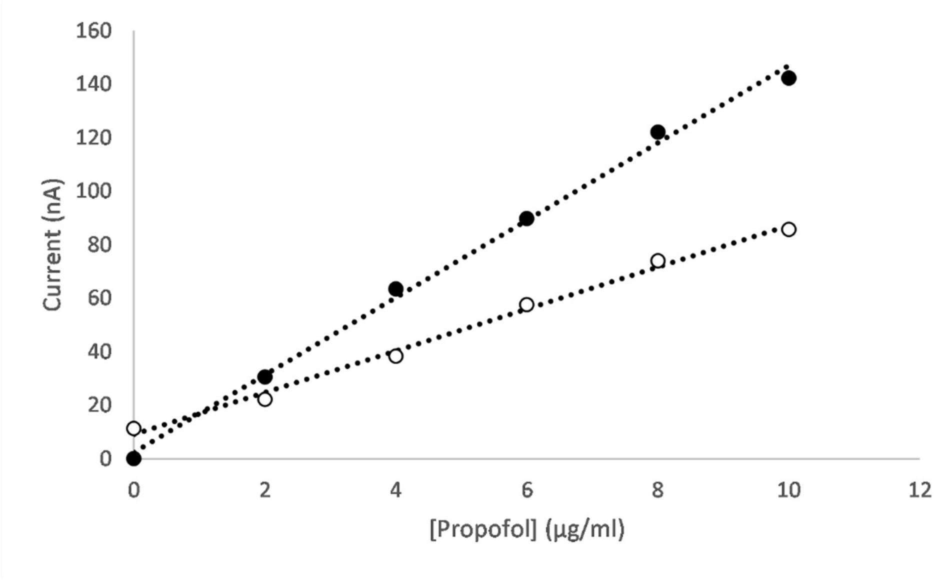

Figure S15 – Current versus propofol concentration for CoO electrode. Black circles - Scan 1, White circles - Scan 2.

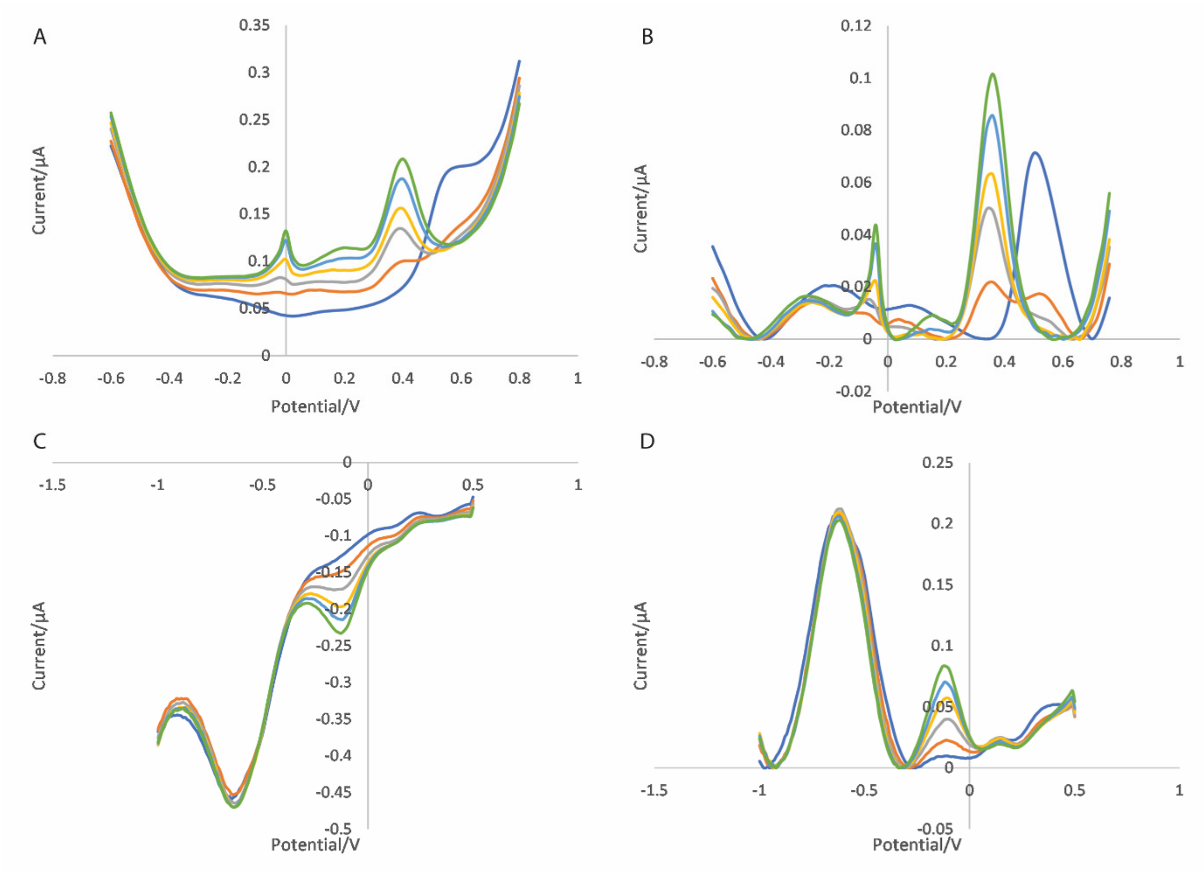

Figure S16 – Differential pulse voltammetry for CuO electrode: A) Scan 1 - raw, B) Scan 1 - baseline corrected, C) Scan 2 - raw, D) Scan 2 - baseline corrected.

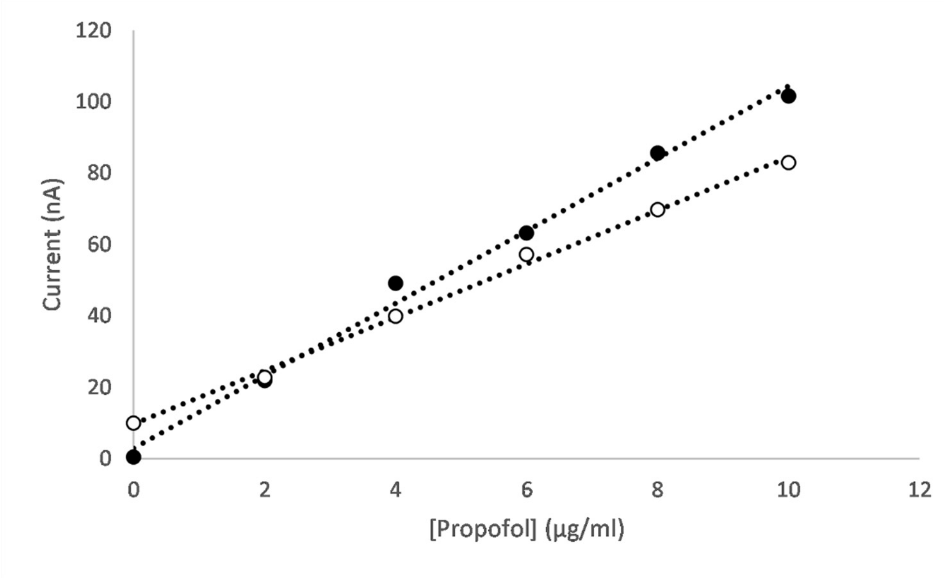

Figure S17 – Current versus propofol concentration for CuO electrode. Black circles - Scan 1, White circles - Scan 2.

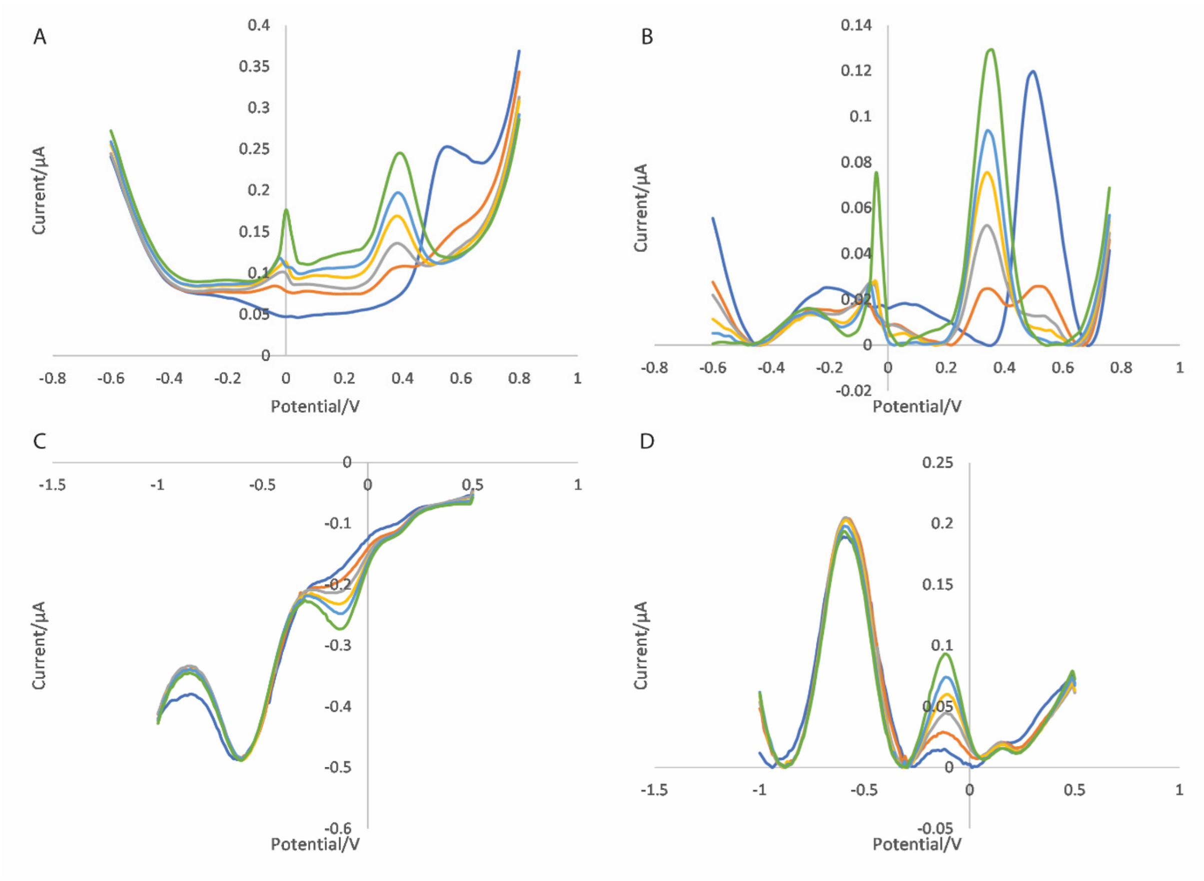

Figure S18 – Differential pulse voltammetry for FeO electrode: A) Scan 1 - raw, B) Scan 1 - baseline corrected, C) Scan 2 - raw, D) Scan 2 - baseline corrected.

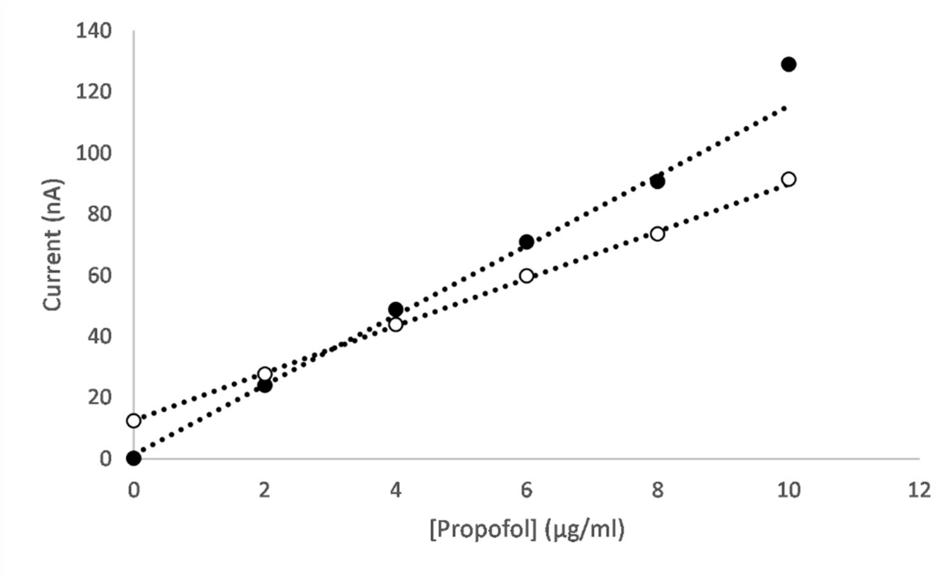

Figure S19 – Current versus propofol concentration for FeO electrode. Black circles - Scan 1, White circles - Scan 2.

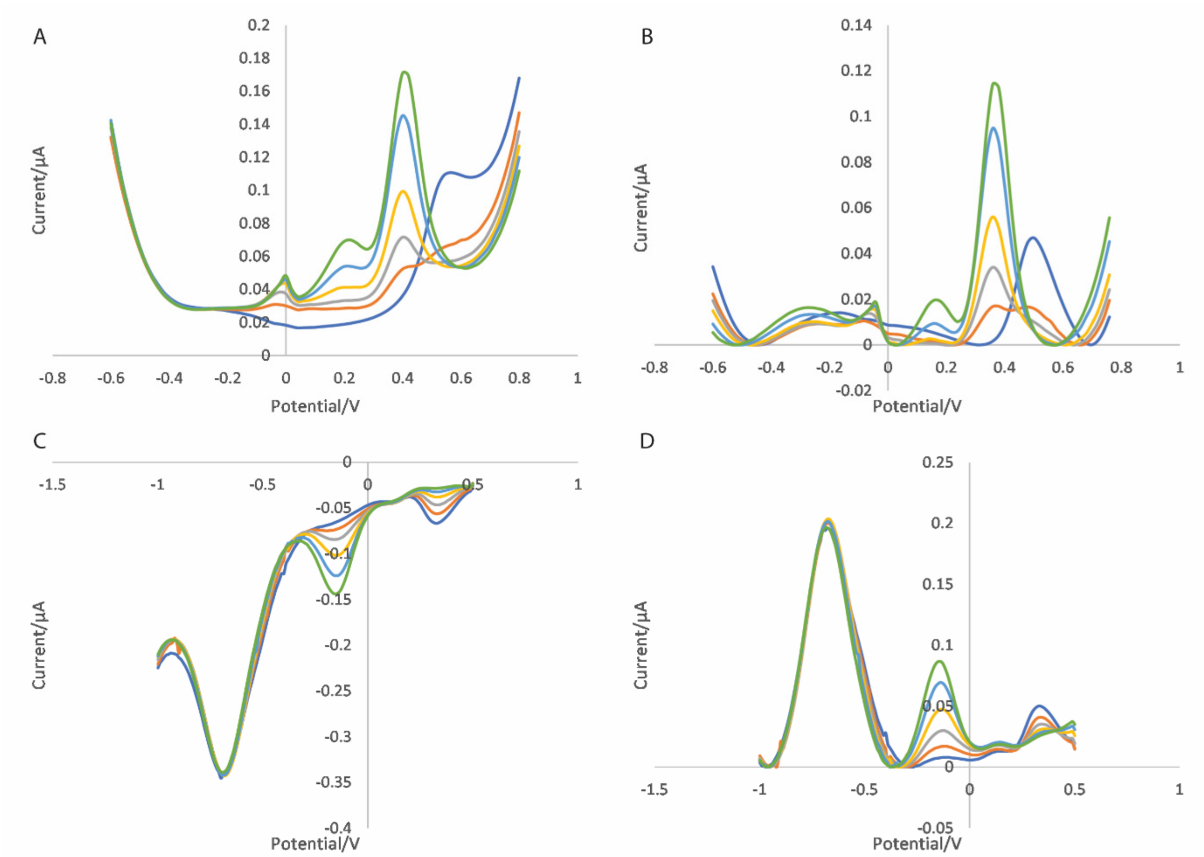

Figure S20 – Differential pulse voltammetry for MnO electrode: A) Scan 1 - raw, B) Scan 1 - baseline corrected, C) Scan 2 - raw, D) Scan 2 - baseline corrected.

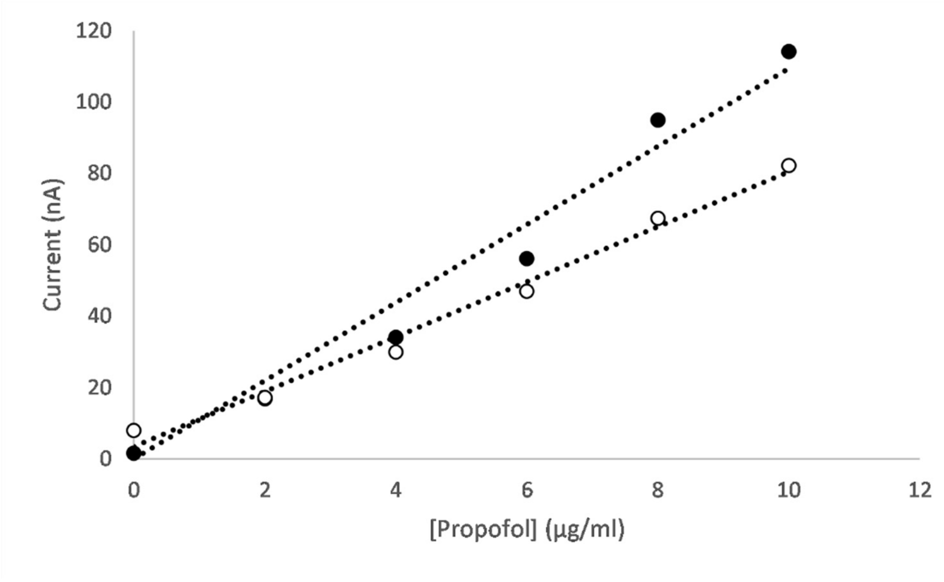

Figure S21 – Current versus propofol concentration for MnO electrode. Black circles - Scan 1, White circles - Scan 2.

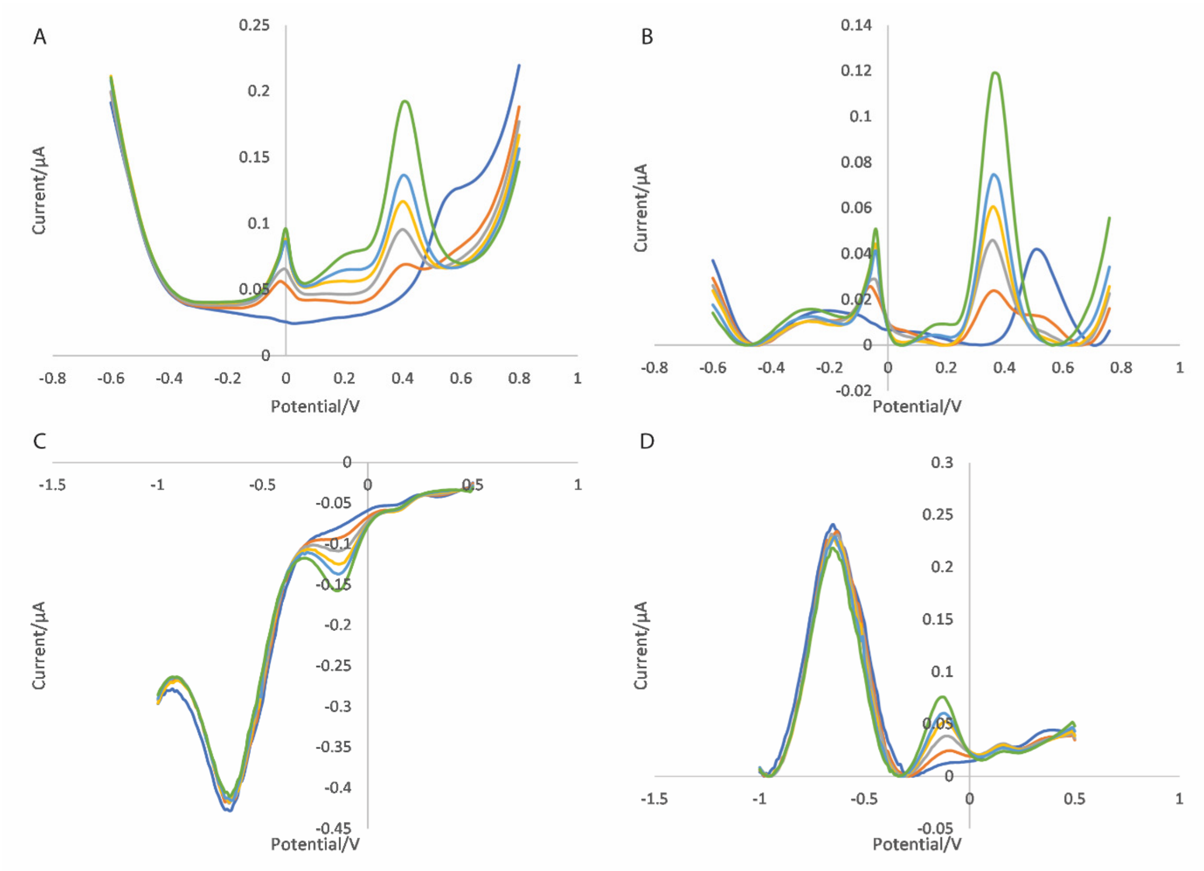

Figure S22 – Differential pulse voltammetry for NiO electrode: A) Scan 1 - raw, B) Scan 1 - baseline corrected, C) Scan 2 - raw, D) Scan 2 - baseline corrected.

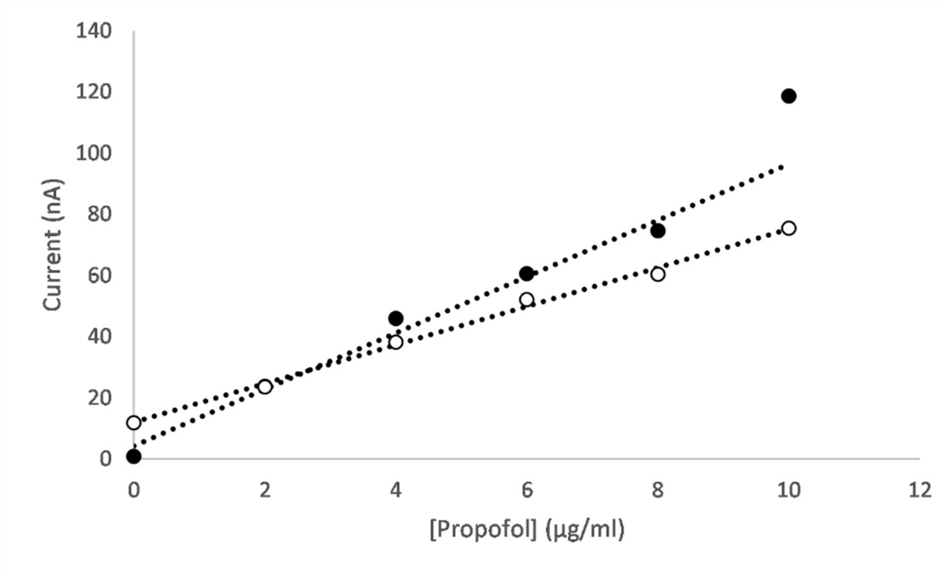

Figure S23 – Current versus propofol concentration for NiO electrode. Black circles - Scan 1, White circles - Scan 2.

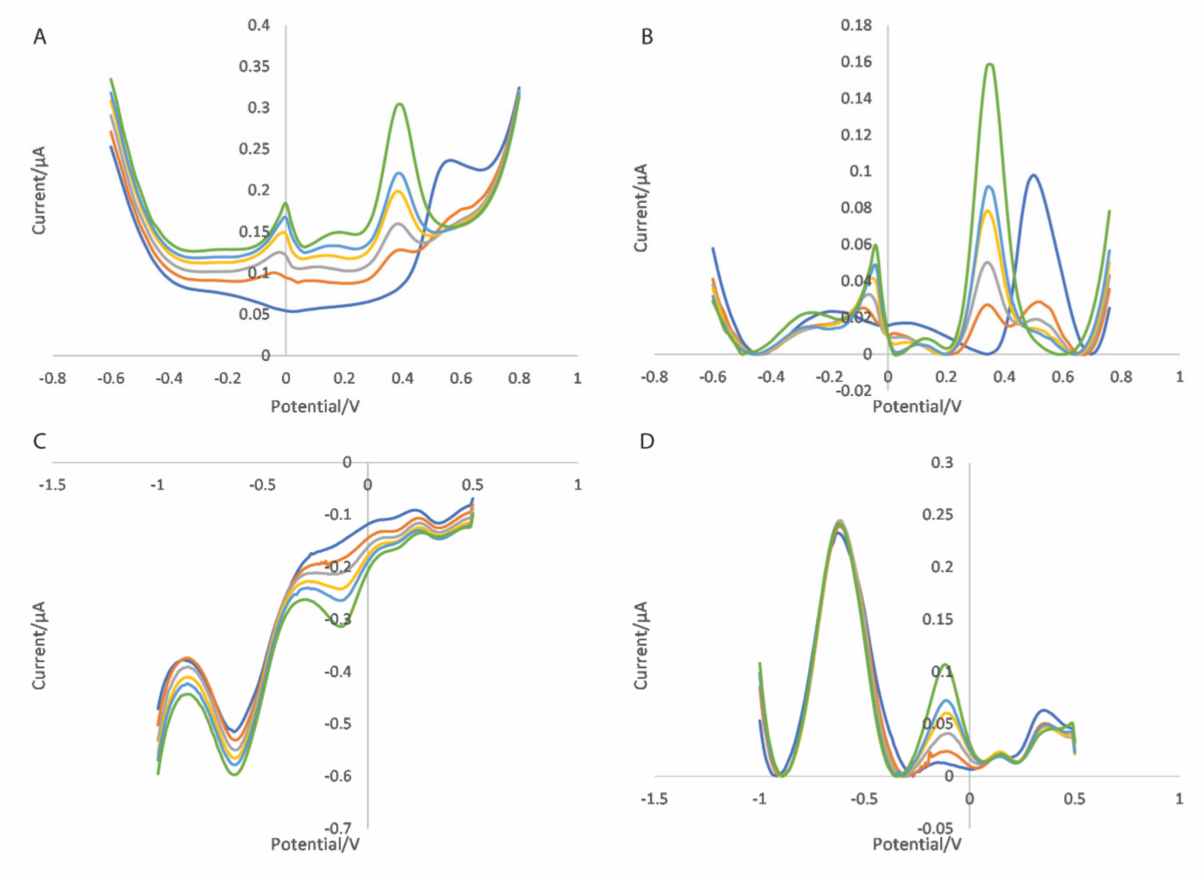

Figure S24 – Differential pulse voltammetry for TiO electrode: A) Scan 1 - raw, B) Scan 1 - baseline corrected, C) Scan 2 - raw, D) Scan 2 - baseline corrected.

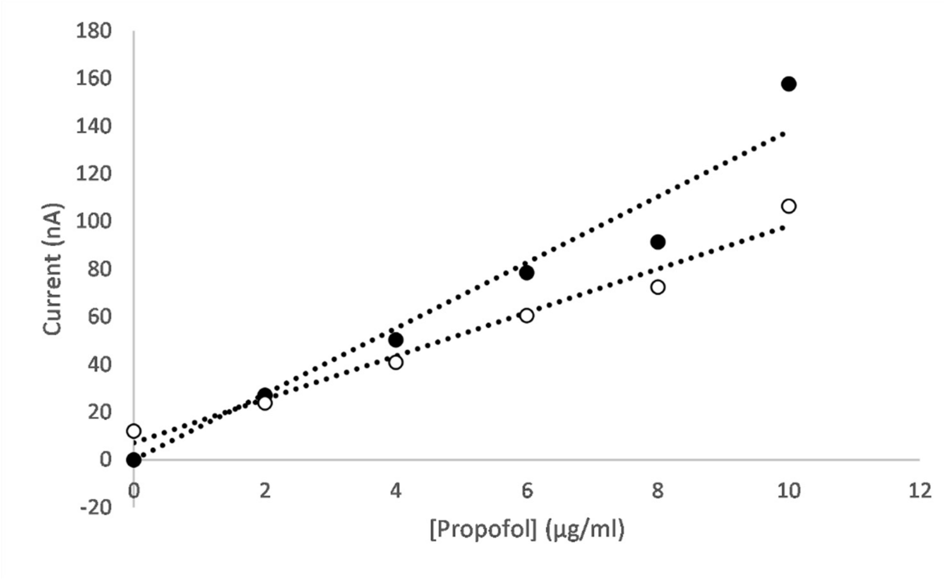

Figure S25 – Current versus propofol concentration forTiO electrode. Black circles - Scan 1, White circles - Scan 2.

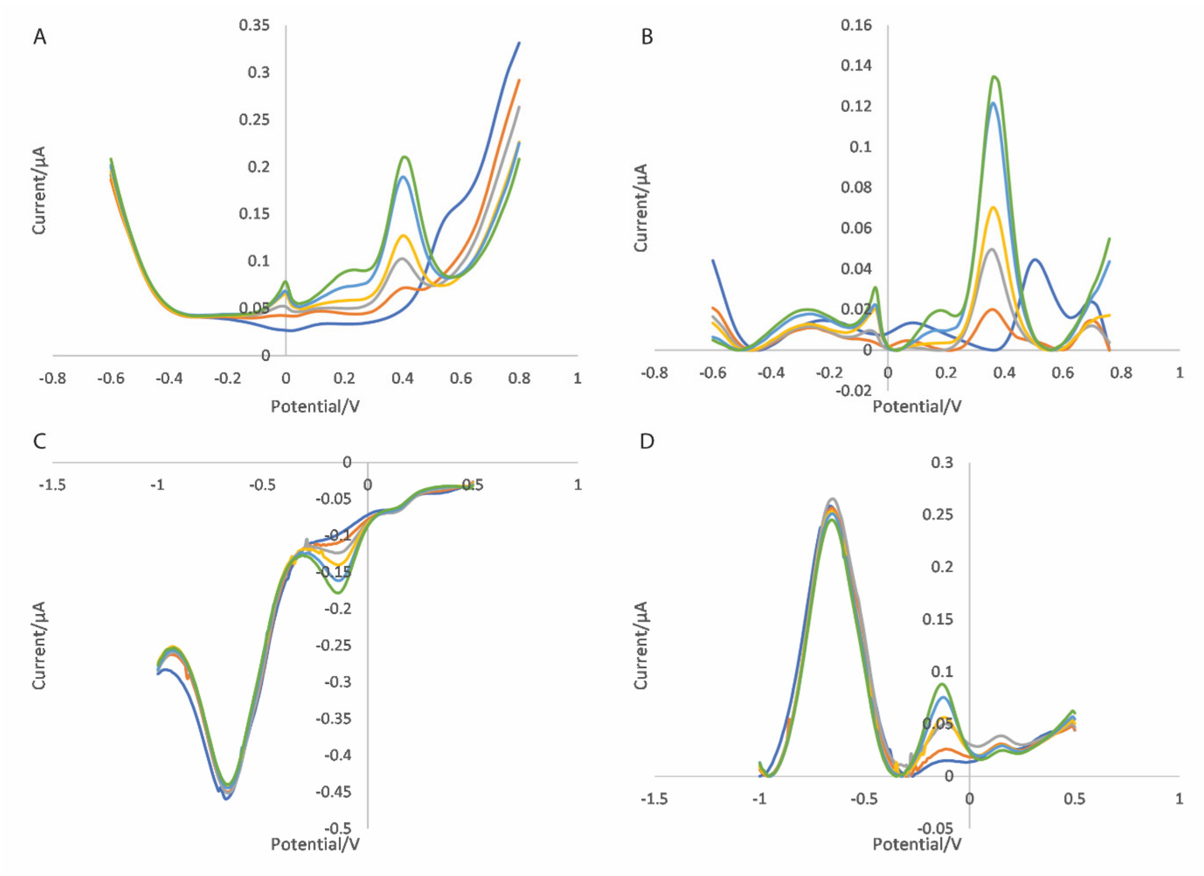

Figure S26 – Differential pulse voltammetry for ZnO electrode: A) Scan 1 - raw, B) Scan 1 - baseline corrected, C) Scan 2 - raw, D) Scan 2 - baseline corrected.

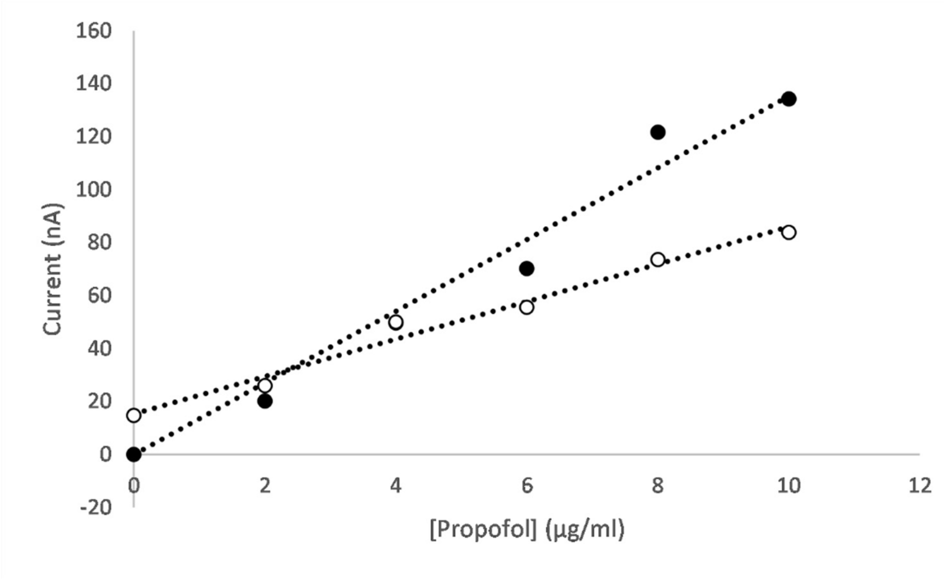

Figure S27 – Current versus propofol concentration for ZnO electrode. Black circles - Scan 1, White circles - Scan 2.

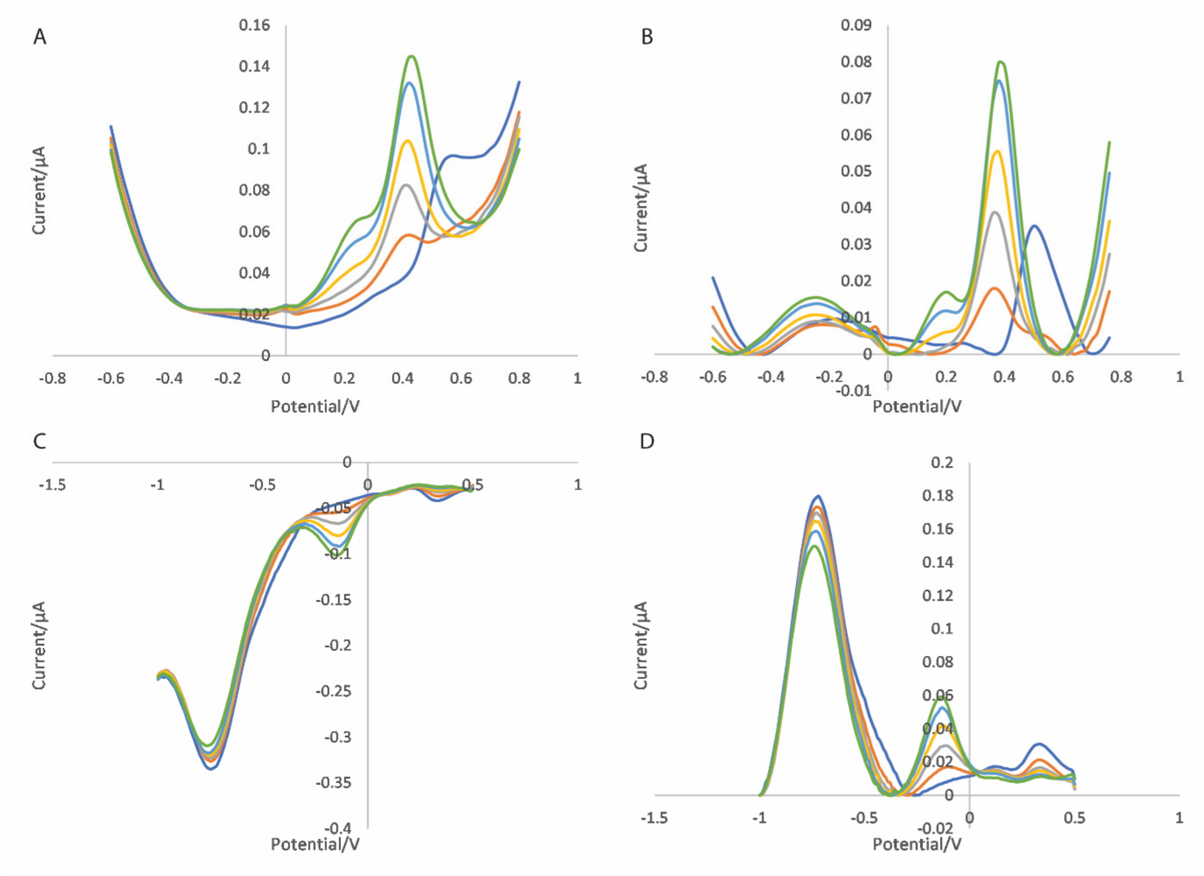

Figure S28 – Differential pulse voltammetry for CoTiO electrode: A) Scan 1 - raw, B) Scan 1 - baseline corrected, C) Scan 2 - raw, D) Scan 2 - baseline corrected.

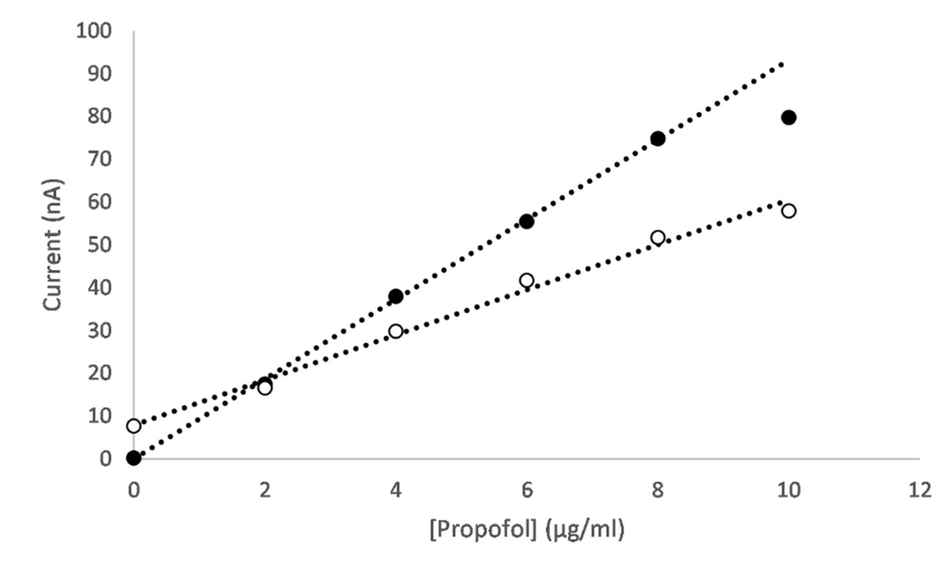

Figure S29 – Current versus propofol concentration for CoTiO electrode. Black circles - Scan 1, White circles - Scan 2.

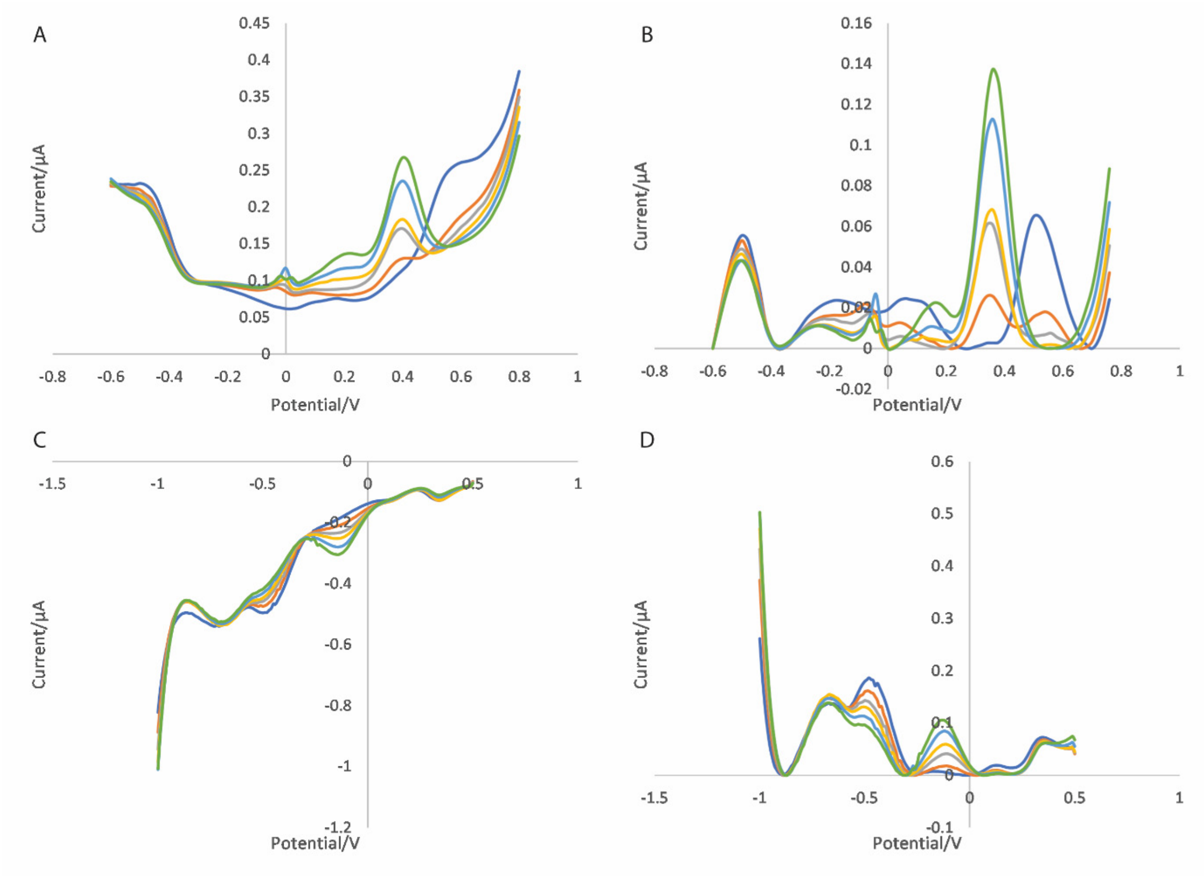

Figure S30 – Differential pulse voltammetry for CuTiO electrode: A) Scan 1 - raw, B) Scan 1 - baseline corrected, C) Scan 2 - raw, D) Scan 2 - baseline corrected.

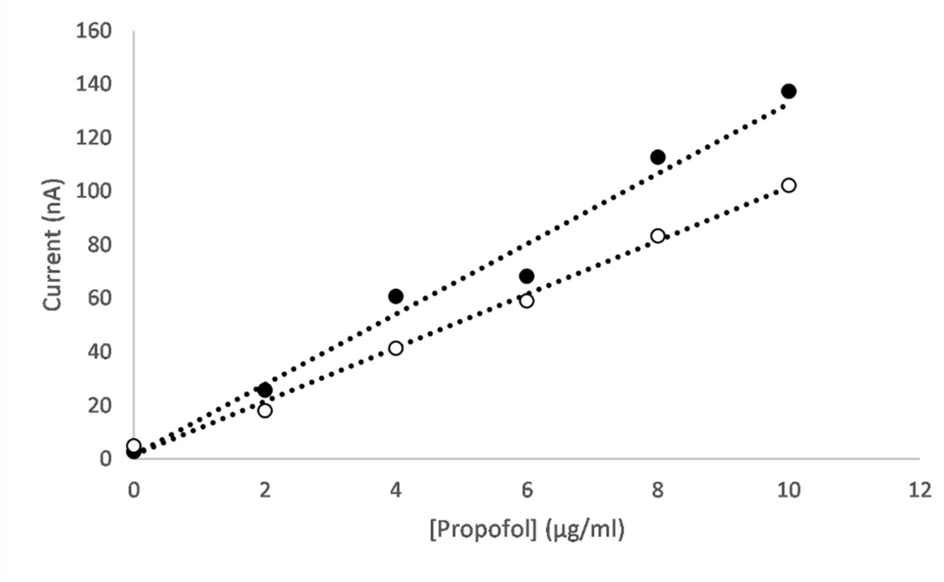

Figure S31 – Current versus propofol concentration for CuTiO electrode. Black circles - Scan 1, White circles - Scan 2.

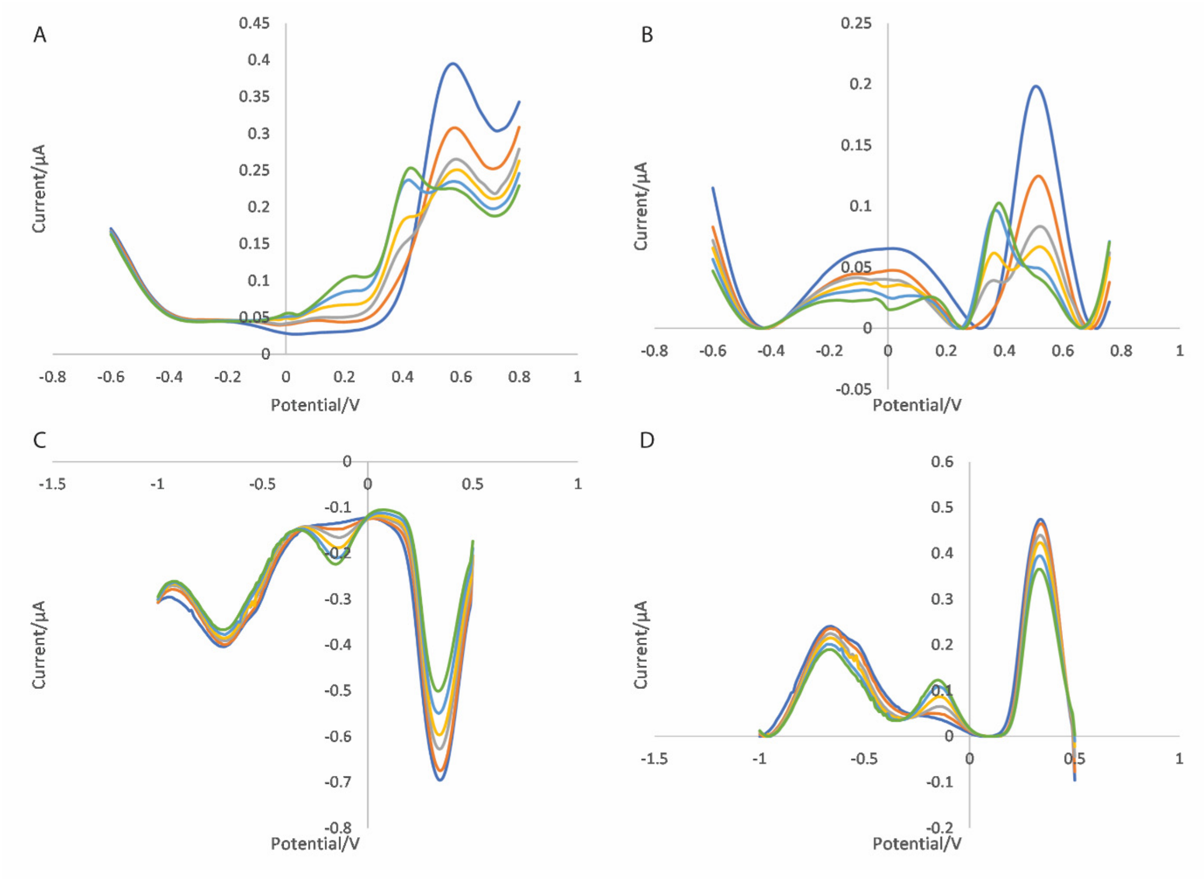

Figure S32 – Differential pulse voltammetry for MnTiO electrode: A) Scan 1 - raw, B) Scan 1 - baseline corrected, C) Scan 2 - raw, D) Scan 2 - baseline corrected.

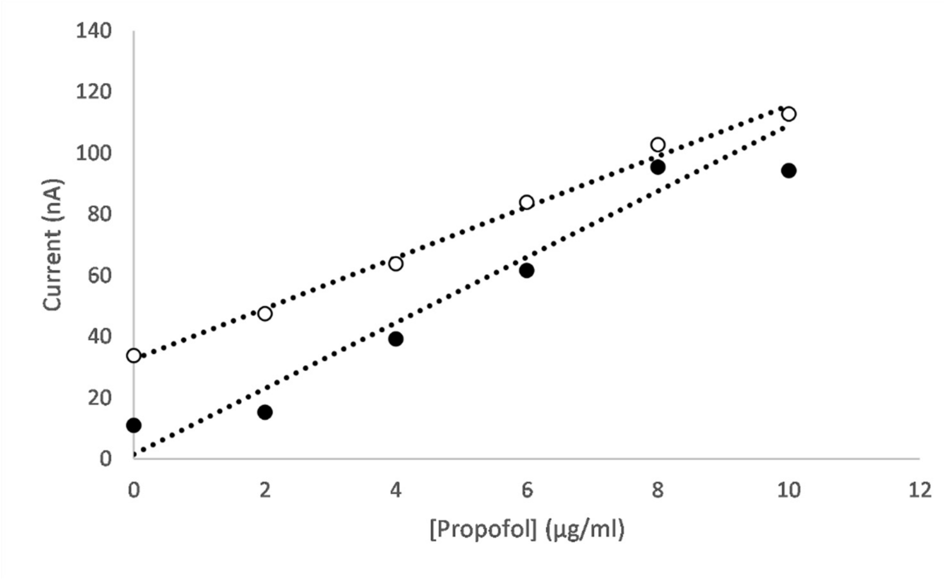

Figure S33 - Current versus propofol concentration for MnTiO electrode. Black circles - Scan 1, White circles - Scan 2.

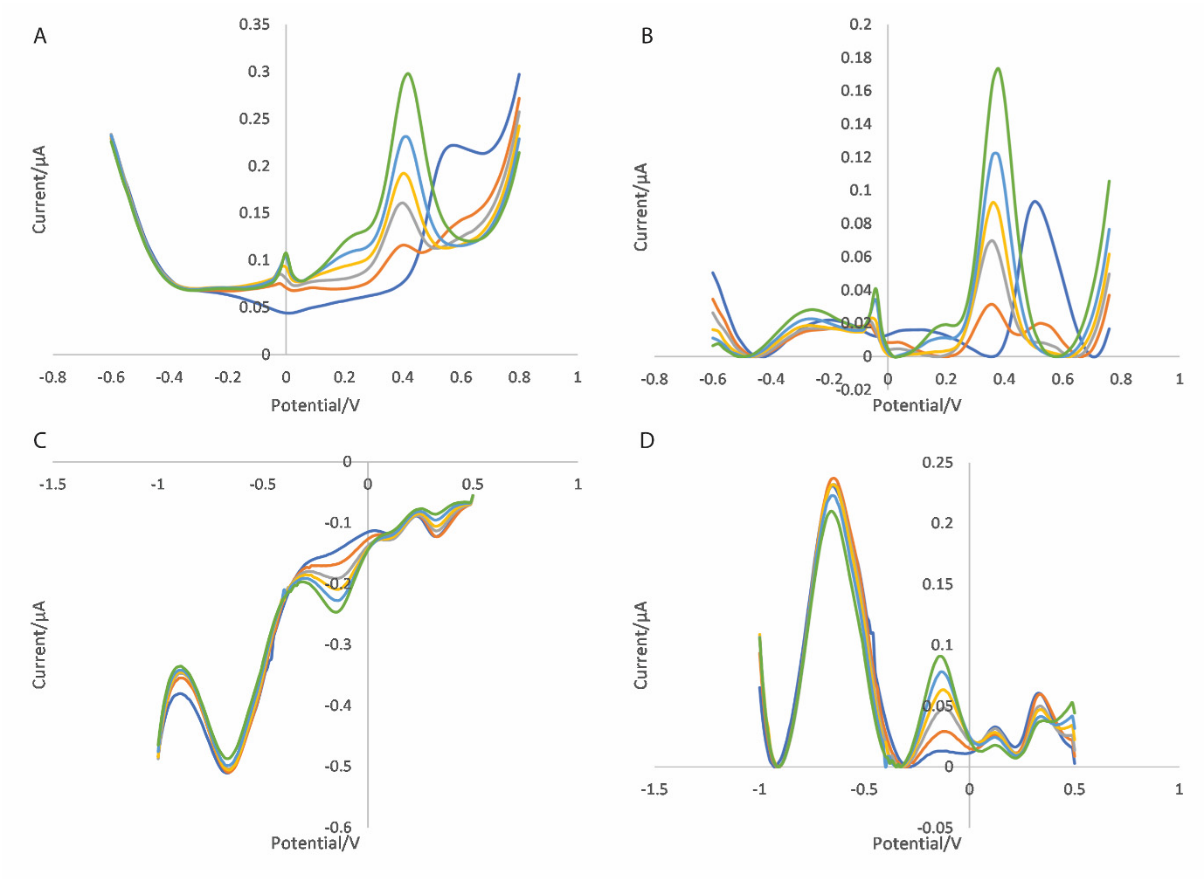

Figure S34 – Differential pulse voltammetry for NiTiO electrode: A) Scan 1 - raw, B) Scan 1 - baseline corrected, C) Scan 2 - raw, D) Scan 2 - baseline corrected.

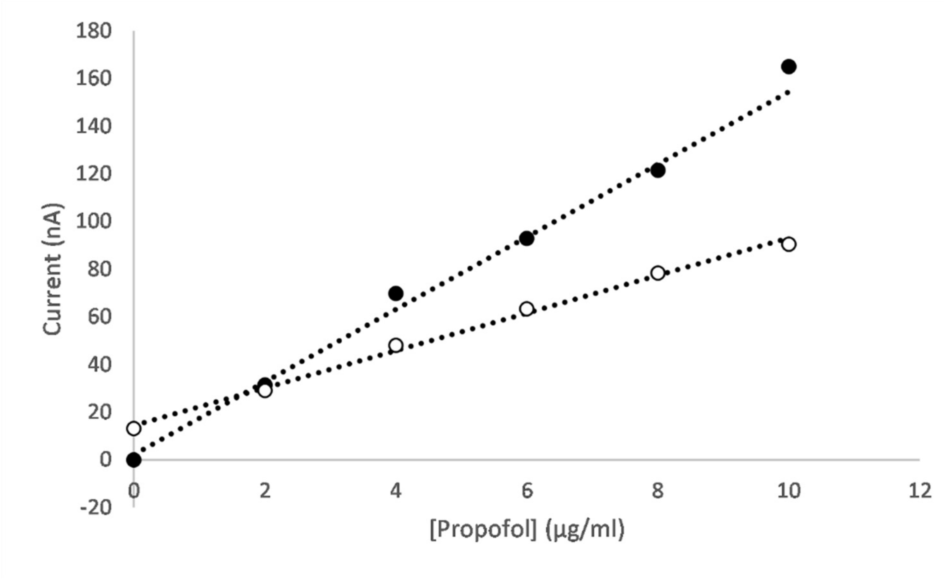

Figure S35 - Current versus propofol concentration for NiTiO electrode. Black circles - Scan 1, White circles - Scan 2.

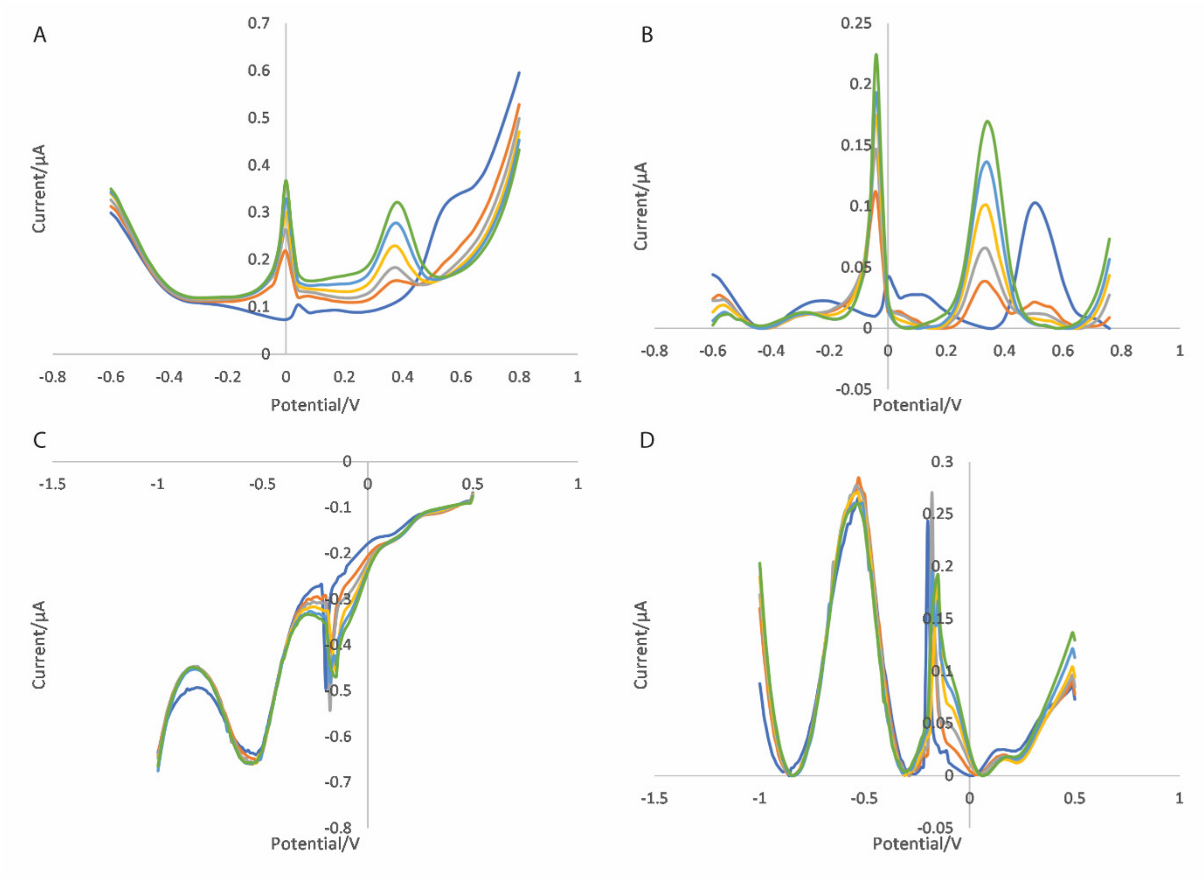

Figure S36 – Differential pulse voltammetry for ZnTiO electrode: A) Scan 1 - raw, B) Scan 1 - baseline corrected, C) Scan 2 - raw, D) Scan 2 - baseline corrected.

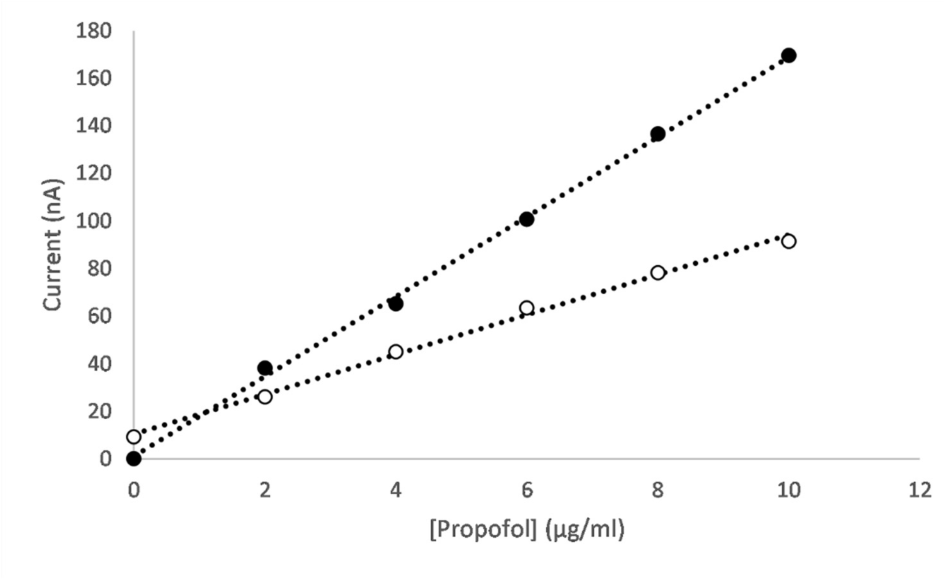

Figure S37 – Current versus propofol concentration for ZnTiO electrode. Black circles - Scan 1, White circles - Scan 2.

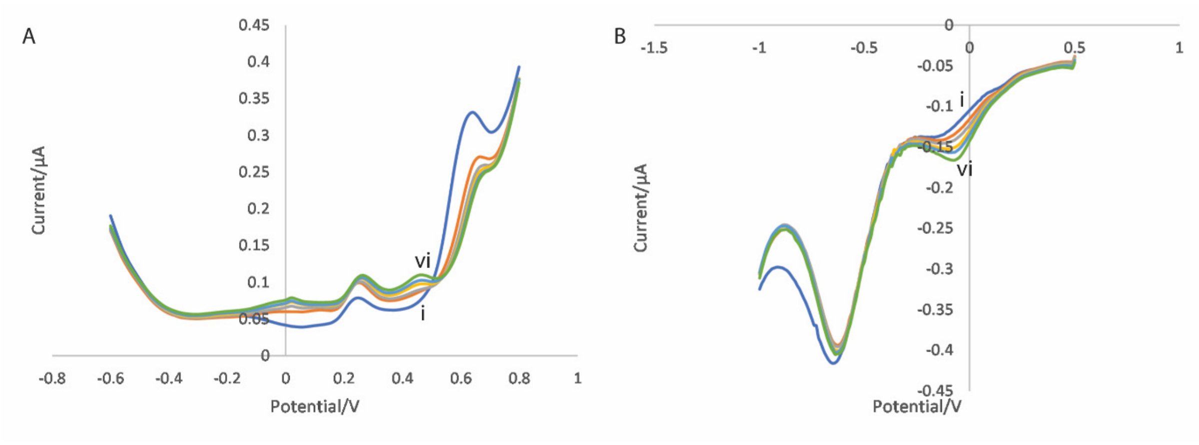

Figure S38 – Differential pulse voltammograms for A) oxidation scan, and B) reduction scan for a CNT/GO/ZnTiONP nanocomposite functionalised electrode in bovine serum with propofol concentrations of i) 0, ii) 2, iii) 4, iv) 6, v) 8 and vi) 10 µg/ml.

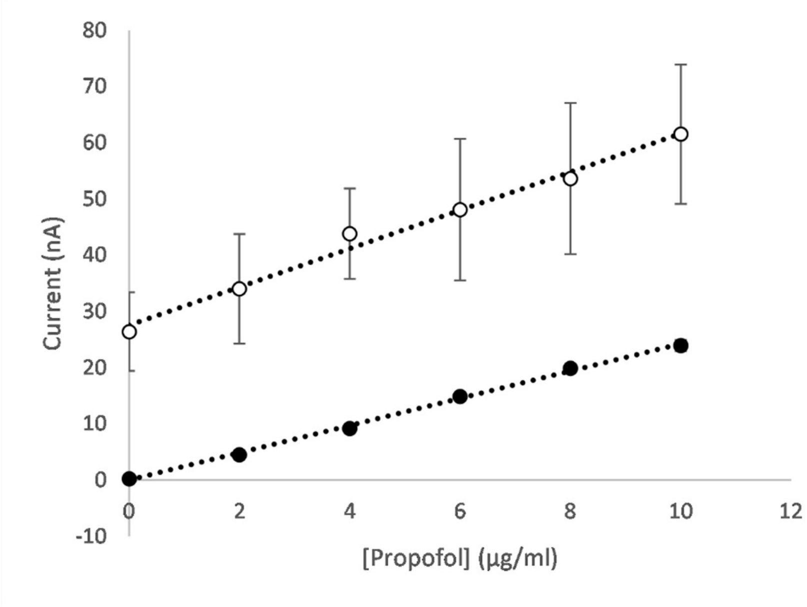

Figure S39 – Peak current versus propofol concentration at +460 mV for the oxidation scan (black circles) and -60 mV for the reduction scan (white circles) for CNT/GO/ZnTiO electrodes in spiked bovine serum. Baseline correction has been applied and the error bars represent one standard deviation (N = 3).

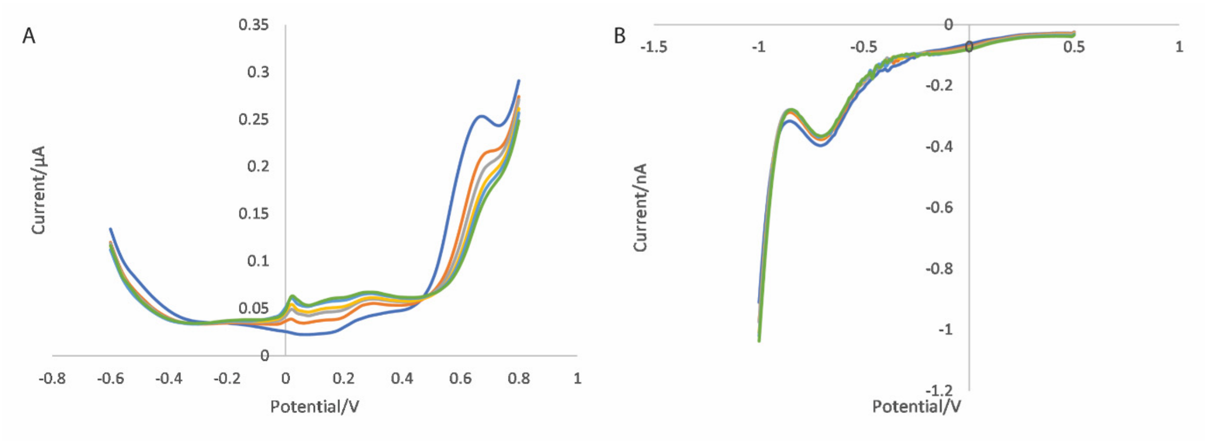

Figure S40 - Differential pulse voltammograms for A) the oxidation scan and B) the reduction scan for ascorbic acid in bovine serum at concentrations of i) 0, ii) 7, iii) 14, iv) 21, v) 28, and vi) 35 µg/ml.
